# Supplementary material for: Germline Variant Burden Warrants Universal Genetic Testing in Pediatric Myeloid Leukemia
Source: medRxiv. 2025 Jul 30:2025.07.29.25332166. Preprint. [Version 1] doi: 10.1101/2025.07.29.25332166 (PMC12324642; doi:10.1101/2025.07.29.25332166)
Supplement: Supplement 1 [file media-1.pdf]

Supplement for Germline Variant Burden Warrants Universal Genetic Testing in Pediatric Myeloid Leukemia

Supplementary Methods: 2
- Whole genome sequencing (WGS) 2
- RNA sequencing 2
- Cytogenetic and structural variants 2
- Statistical Methods 2

Supplementary Figures 3
- Supplementary Figure 1: Cohort Demographics 3
- Supplementary Figure 2: Flowchart of programmatic filtering of variants to be hand-curated 4
- Supplementary Figure 3: Demographics of germline variant carriers 5
- Supplementary Figure 4: Oncoplot of germline and somatic variants 6
- Supplementary Figure 5: Allelic bias in P/LP variants 7
- Supplementary Figure 6: Number of variants in selected genes for 1000 Genomes Control subjects as compared to pediatric AML patients 8

Supplementary Tables 9
- Supplementary Table 1: Cohort Demographics 9
- Supplementary Table 2: Target genes associated with hematologic malignancies, cancer risk, immunodeficiencies, bone marrow, or blood diseases and genomic GRCh38 coordinates 10
- Supplementary Table 3: P/LP variants with moderate evidence for association to hematopoietic malignancy risk 28
- Supplementary Table 4: P/LP variants in genes with limited evidence for association to hematopoietic malignancy risk 30
- Supplementary Table 5: P/LP variants with limited evidence for association to solid tumor risk 31
- Supplementary Table 6: Heterozygous P/LP variants in genes with autosomal recessive hematopoietic malignancy risk inheritance 32
- Supplementary Table 7: Heterozygous P/LP variants in genes with autosomal recessive solid tumor risk inheritance 33
- Supplementary Table 8: Heterozygous P/LP variants in genes associated with other hematopoietic diseases or developmental disorders with autosomal recessive inheritance 35
- Supplementary Table 9: P/LP variants in genes with a known somatic role in AML but no prior evidence for germline risk to malignancy 36
- Supplementary Table 10: Patients with multiple P/LP variants 38
- Supplementary Table 11: Additional information for P/LP variants 39
- Supplementary Table 12: Additional somatic variants in genes with P/LP variants 48
- Supplementary Table 13: Genes and mode of inheritance included in cohort meta-analysis 48

Supplementary References: 52

Supplementary Methods:

Whole genome sequencing (WGS)

Whole-genome DNA sequencing was conducted as described previously[1]. Briefly, paired-end libraries were prepared using the Illumina library construction protocol and sequenced with 30x average coverage on Illumina HiSeq2500 instruments. Reads were mapped to GRCh37 (hg19) using bwa[2]; SNVs and indel variants were called with *Bambino*[3]. WGS for TARGET-21, previously aligned to GRCh38, was retrieved with BamSliceR[4].

RNA sequencing

Transcriptome data were generated by the British Columbia Genome Sciences Center (BCGSC; Vancouver, BC). Total RNA was ribo-depleted and reverse transcribed in a strand-specific mRNA library construction protocol, indexed, pooled, and sequenced on an Illumina HiSeq 2500 as previously published[5] to produce 75 base-pair, paired-end sequence reads. Sequencing data were aligned to human genome assembly GRCh38. Variants in regions of interest were identified using BamSliceR[4].

Cytogenetic and structural variants

Karyotypes were centrally reviewed at COG. Somatic structural variants for comparison between groups were called from WGS data using Manta with default parameters for somatic structural variant detection[6]. Suspected driver lesions were orthogonally validated as previously published.[7]

Statistical Methods

All statistical analyses were performed using R version 4.3.2. Figures were generated using ggplot21. Pathway analyses were performed using clusterProfiler[8]. Poisson goodness of fit tests were performed using vcd (visualizing categorical data)[9]. The oncoplot of variants was generated using maftools[10].

Supplementary Figures

Supplementary Figure 1: Cohort Demographics.

A) Ethnic classifications. B) Scatter plot of the distribution of ages. C) Sex. D) Major somatic driver groups.

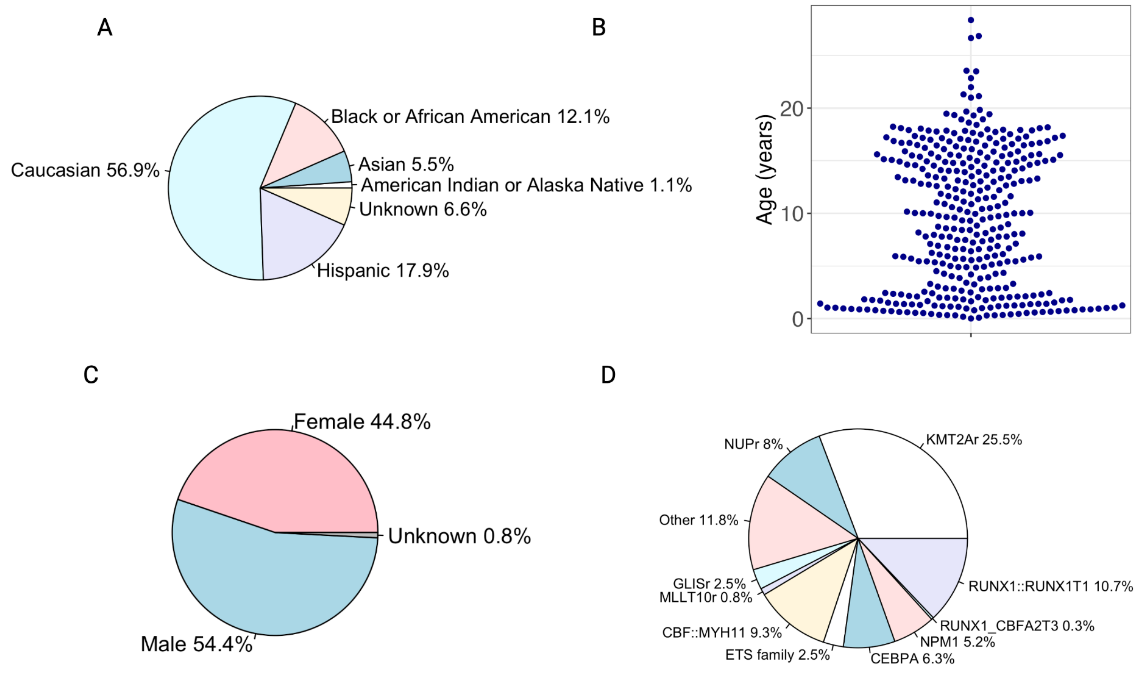

Supplementary Figure 2: Flowchart of programmatic filtering of variants to be hand-curated.

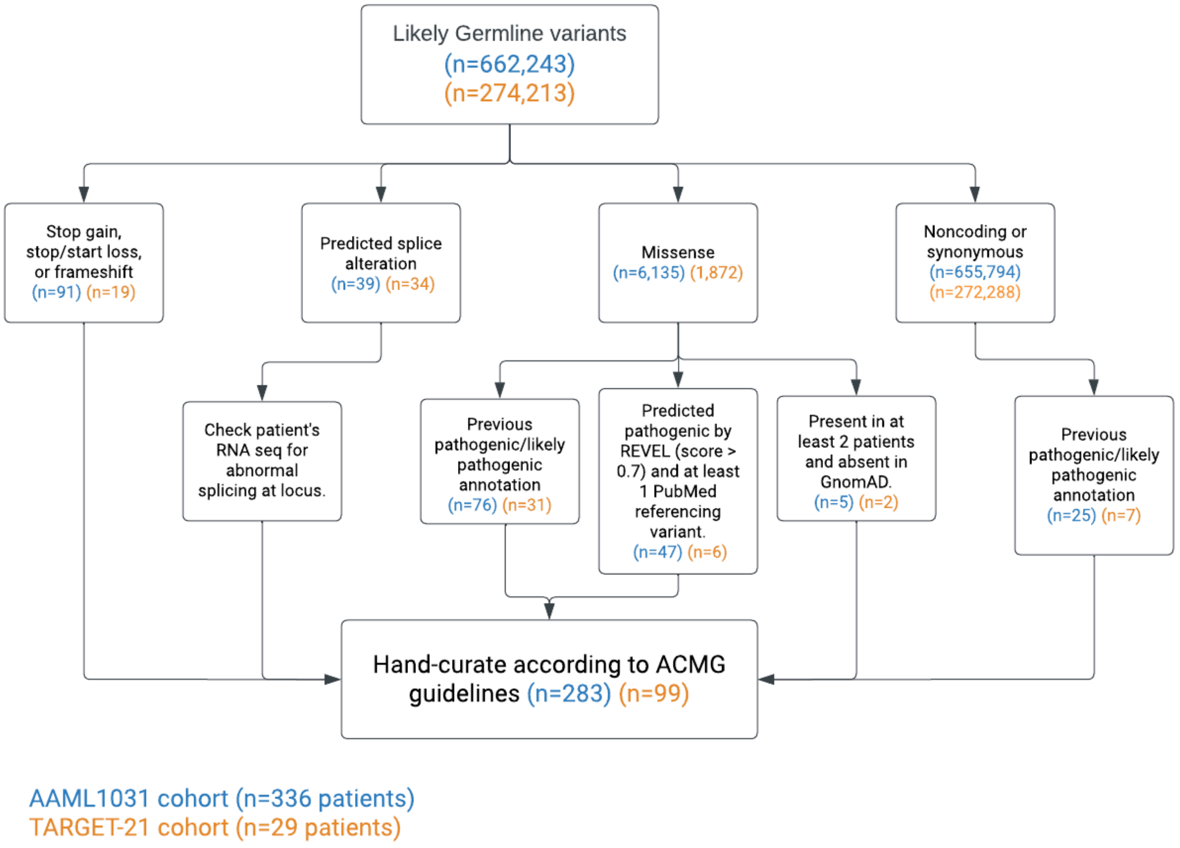

Supplementary Figure 3: Demographics of germline variant carriers.

A-D) Comparison of patients carrying a germline variant versus patients without a germline variant. A) Distribution of ages, B) Sex, C) Ethnic classification, D) AML somatic driver group and E) Event-free survival.

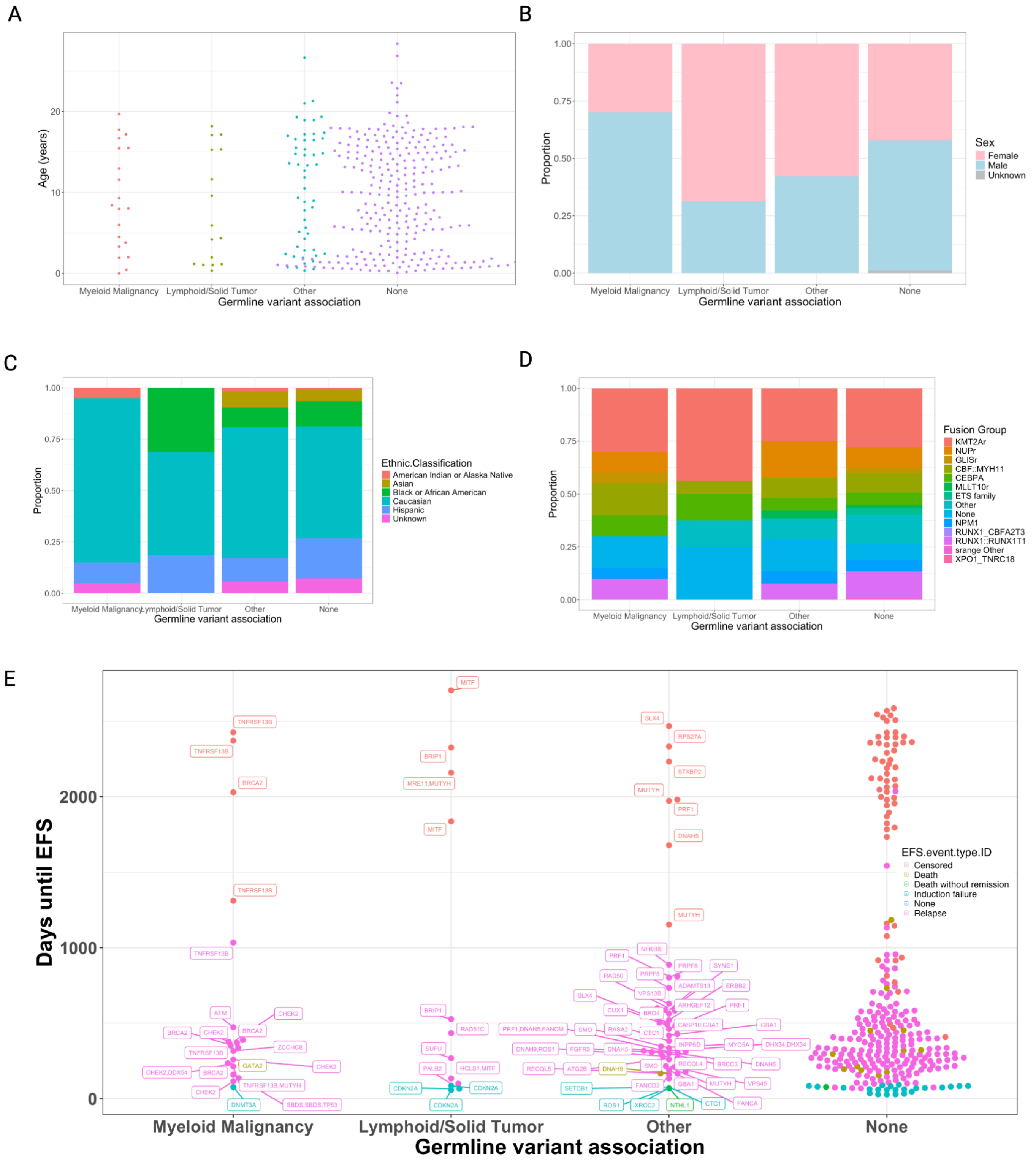

Supplementary Figure 4: Oncoplot of germline and somatic variants.

Columns represent patients. Green = germline variant calls, blue = somatic variants (covariates), red = somatic fusions (covariates), yellow = multiple variants.

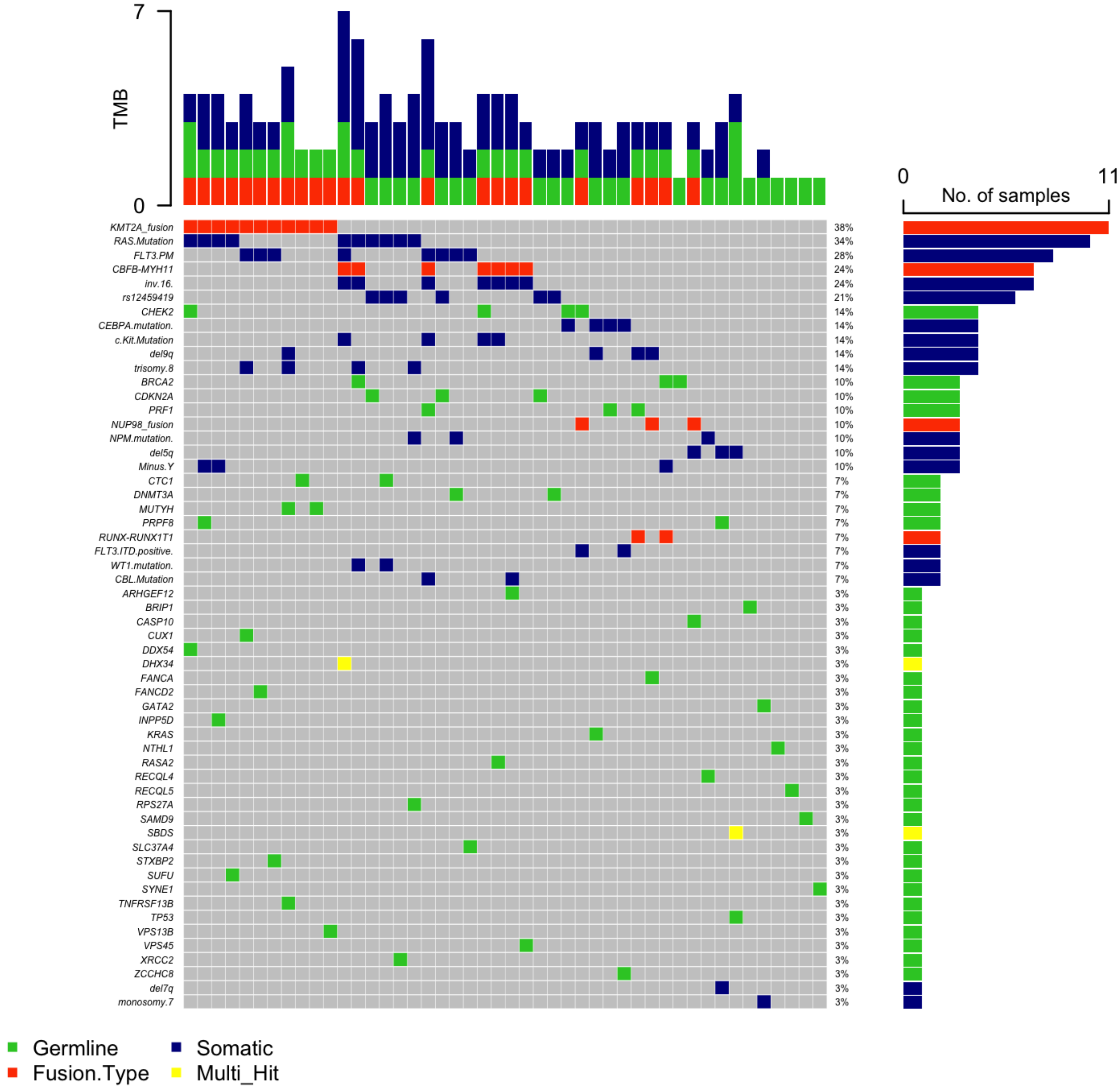

Supplementary Figure 5: Allelic bias in P/LP variants

A) P/LP variants and B) predicted nonsense-mediated mRNA decay (NMD) P/LP variants. VAF=variant allele frequency.

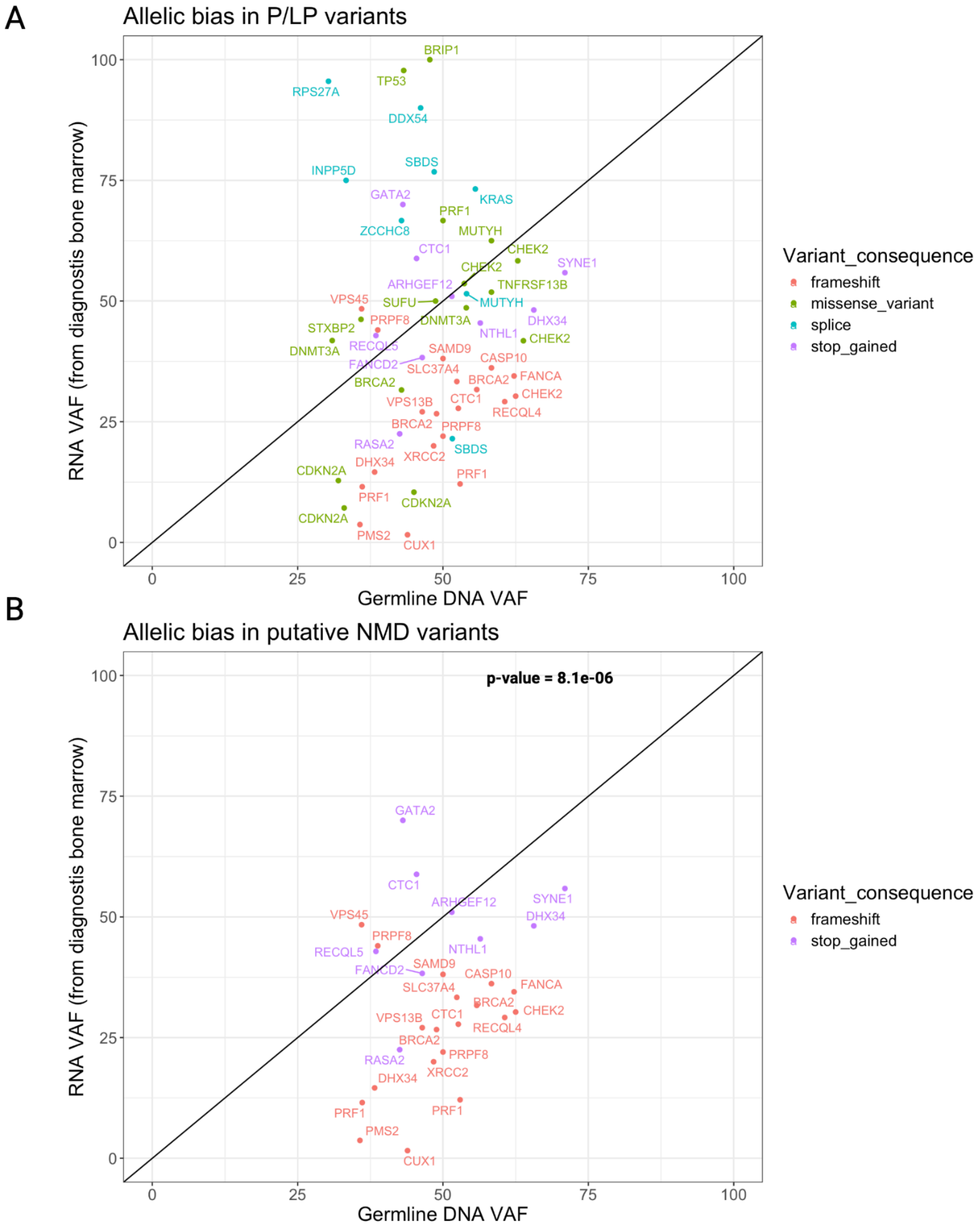

Supplementary Figure 6: Number of variants in selected genes for 1000 Genomes Control subjects as compared to pediatric AML patients

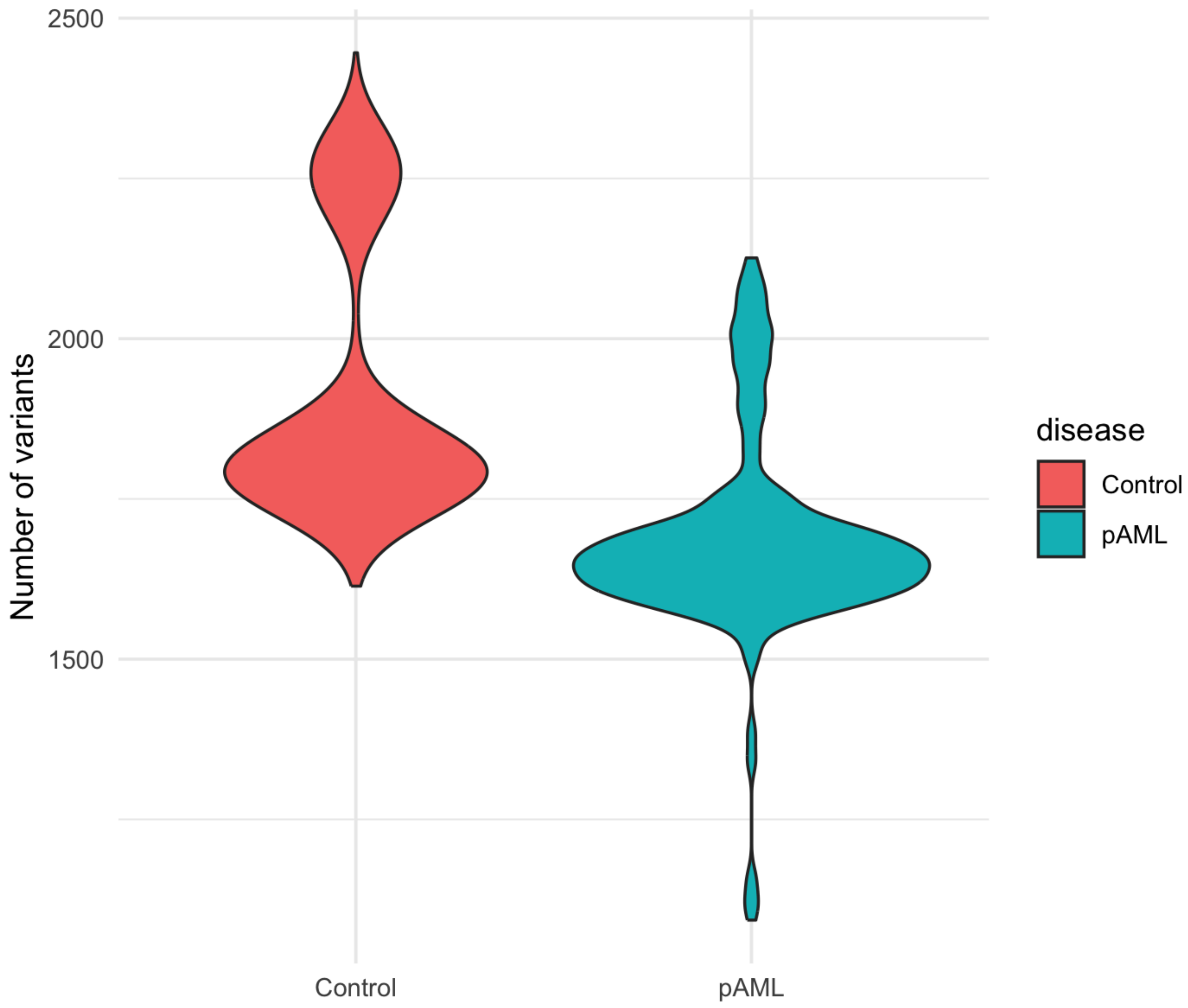

Supplementary Tables

Supplementary Table 1: Cohort Demographics

	AAML03P1 (N=4)	AAML0531 (N=25)	AAML1031 (N=335)	Overall (N=364)	p
**AgeGroup**					
AYA	0 (0%)	4 (16.0%)	90 (26.9%)	94 (25.8%)	0.559
Child	3 (75.0%)	16 (64.0%)	152 (45.4%)	171 (47.0%)	
Infant	1 (25.0%)	5 (20.0%)	90 (26.9%)	96 (26.4%)	
Missing	0 (0%)	0 (0%)	3 (0.9%)	3 (0.8%)	
**Sex**					
Female	1 (25.0%)	10 (40.0%)	154 (46.0%)	165 (45.3%)	0.799
Male	3 (75.0%)	15 (60.0%)	181 (54.0%)	199 (54.7%)	
**Fusion Group**					
CBF::MYH11	0 (0%)	0 (0%)	34 (10.1%)	34 (9.3%)	<0.001
CEBPA	0 (0%)	0 (0%)	23 (6.9%)	23 (6.3%)	
ETS family	0 (0%)	0 (0%)	9 (2.7%)	9 (2.5%)	
GLISr	0 (0%)	0 (0%)	9 (2.7%)	9 (2.5%)	
KMT2Ar	0 (0%)	2 (8.0%)	101 (30.1%)	103 (28.3%)	
MLLT10r	1 (25.0%)	2 (8.0%)	3 (0.9%)	6 (1.6%)	
NPM1	0 (0%)	0 (0%)	19 (5.7%)	19 (5.2%)	
NUP98 NSD1/HOX	2 (50.0%)	5 (20.0%)	16 (4.8%)	23 (6.3%)	
NUP98 reader	1 (25.0%)	0 (0%)	6 (1.8%)	7 (1.9%)	
RUNX1::RUNX1T1	0 (0%)	1 (4.0%)	39 (11.6%)	40 (11.0%)	
Other	0 (0%)	2 (8.0%)	53 (15.8%)	55 (15.1%)	
None	0 (0%)	13 (52.0%)	23 (6.9%)	36 (9.9%)	
**Ethnic Classification**					
Black or African American	1 (25.0%)	3 (12.0%)	40 (11.9%)	44 (12.1%)	1
Caucasian	3 (75.0%)	15 (60.0%)	189 (56.4%)	207 (56.9%)	
Asian	0 (0%)	2 (8.0%)	18 (5.4%)	20 (5.5%)	
Hispanic	0 (0%)	4 (16.0%)	61 (18.2%)	65 (17.9%)	
Unknown	0 (0%)	1 (4.0%)	23 (6.9%)	24 (6.6%)	
American Indian or Alaska Native	0 (0%)	0 (0%)	4 (1.2%)	4 (1.1%)	

Supplementary Table 2: Target genes associated with hematologic malignancies, cancer risk, immunodeficiencies, bone marrow, or blood diseases and genomic GRCh38 coordinates.

Coordinates included all coding regions and untranslated regions (promoter, introns, 5’ and 3’ untranslated regions).

**SYMBOL**	**Coordinates (GRCh38)**
ABL1	9:130713043-130887675
ABL2	1:179099330-179229684
ACD	16:67657512-67660810
ACTB	7:5526409-5563902
ACTN1	14:68874128-68979440
ADA	20:44584896-44652252
ADA2	22:17178790-17258235
ADAMTS13	9:133414358-133459402
ADD3	10:109996368-110135565
AIP	11:67468174-67491154
AIRE	21:44285838-44298648
AK2	1:33007986-33080996
AKT1	14:104769349-104795759
ALAS2	X:55009055-55030977
ALK	2:29192774-29921586
ANKRD26	10:26973793-27100494
AP3B1	5:78000522-78294762
APC	5:112707518-112846239
ARHGEF12	11:120336413-120489937
ARID1A	1:26693236-26782104
ARID1B	6:156776020-157210779
ARID2	12:45729706-45908040
ASXL1	20:32358330-32439319
ASXL2	2:25733753-25878487
ATF7IP	12:14365676-14502931
ATG2B	14:96279195-96363341
ATM	11:108222804-108369102

ATR	3:142449007-142578733
ATRX	X:77504880-77786233
AXIN2	17:65528563-65561648
BAP1	3:52401008-52410008
BARD1	2:214725646-214809683
BCL10	1:85265776-85276632
BCL11B	14:99169287-99272197
BCL2	18:63123346-63320128
BCL6	3:187721377-187745725
BCOR	X:40049815-40177329
BCORL1	X:129981107-130058071
BIRC3	11:102317484-102339403
BIRC6	2:32357023-32619571
BLM	15:90717346-90816166
BLOC1S3	19:45178784-45216933
BLOC1S6	15:45587214-45615945
BMPR1A	10:86755786-86932825
BRAF	7:140719327-140924929
BRCA1	17:43044295-43170245
BRCA2	13:32315086-32400268
BRCC3	X:155071420-155123077
BRD4	19:15235519-15332545
BRINP3	1:190097658-190478404
BRIP1	17:61679139-61863559
BTG1	12:92140278-92145846
BTK	X:101349338-101390796
BUB1B	15:40161023-40221123
CALR	19:12938578-12944489
CAMTA1	1:6785454-7769706
CARD11	7:2906142-3044228
CASP10	2:201182872-201229428
CASP8	2:201233443-201361836

CBFA2T3	16:88874858-88977207
CBFB	16:67028984-67101058
CBL	11:119206298-119313926
CBLB	3:105655461-105869552
CBLC	19:44777869-44800652
CCND1	11:69641156-69654474
CCND3	6:41934934-42050357
CCR4	3:32951644-32956349
CD27	12:6444955-6451718
CD40LG	X:136648158-136660390
CD70	19:6583183-6604103
CD79A	19:41877279-41881372
CD79B	17:63928738-63932336
CDAN1	15:42723544-42737128
CDC73	1:193121983-193254815
CDH1	16:68737292-68835537
CDH11	16:64943753-65126112
CDIN1	15:36579626-36810248
CDK4	12:57747727-57756013
CDKN1A	6:36676460-36687337
CDKN1B	12:12685498-12722369
CDKN1C	11:2883213-2885775
CDKN2A	9:21967752-21995301
CDKN2C	1:50960745-50974634
CEBPA	19:33299934-33302534
CEP57	11:95789965-95832693
CHD2	15:92886203-93027996
CHD4	12:6570082-6614524
CHEK2	22:28687743-28742422
CLPB	11:72285495-72434680
CREBBP	16:3725054-3880713
CRLF2	Y:1190490-1212723, X:1190490-1212723

CSF1R	5:150053291-150113372
CSF2RA	Y:1268800-1310381, X:1268800-1310381
CSF3R	1:36466043-36483278
CTC1	17:8224815-8248058
CTCF	16:67562467-67639177
CTLA4	2:203853888-203873965
CTSC	11:88265069-88359684
CUX1	7:101815904-102283958
CXCR2	2:218125289-218137251
CXCR4	2:136114349-136119177
CYCS	7:25118656-25125260
CYLD	16:50742050-50801935
DAXX	6:33318558-33323016
DDB2	11:47214465-47239217
DDX41	5:177511577-177516961
DDX54	12:113157173-113185479
DHX15	4:24517441-24584554
DHX29	5:55256055-55307694
DHX34	19:47349315-47382704
DICER1	14:95086228-95158010
DIS3	13:72752169-72782096
DIS3L2	2:231961245-232344350
DKC1	X:154762742-154777689
DNAH5	5:13690328-14011818
DNAH9	17:11598470-11969748
DNAJC21	5:34929559-34958964
DNM2	19:10718055-10833488
DNMT1	19:10133342-10231286
DNMT3A	2:25227855-25342590
DOCK8	9:214854-465259
DROSHA	5:31400494-31532196
DTNBP1	6:15522807-15663058

DTX1	12:113056730-113098028
EBF1	5:158695920-159099916
EED	11:86201212-86278813
EFL1	15:82130206-82262773
EGFR	7:55019017-55211628
ELANE	19:851014-856247
EP300	22:41092510-41180077
EPCAM	2:47345158-47387601
EPOR	19:11377207-11384342
ERBB2	17:39687914-39730426
ERBB4	2:211375717-212538841
ERCC1	19:45407334-45478828
ERCC2	19:45349837-45370918
ERCC3	2:127257290-127294166
ERCC4	16:13920138-13952348
ERCC5	13:102845831-102875995
ERCC6L2	9:95871264-96121154
ERG	21:38380027-38661780
ETNK1	12:22625075-22690665
ETV6	12:11649674-11895377
EXO1	1:241847967-241895148
EXT1	8:117794490-118111826
EXT2	11:44095648-44251962
EZH2	7:148807257-148884321
FADD	11:70203296-70207390
FAM111B	11:59107185-59127412
FAM47A	X:34129752-34132314
FANCA	16:89726683-89816977
FANCB	X:14690388-14873255
FANCC	9:95099054-95426796
FANCD2	3:10026370-10101932
FANCE	6:35452338-35467104

FANCF	11:22622533-22625823
FANCG	9:35073835-35080004
FANCI	15:89243945-89317261
FANCL	2:58159243-58241410
FANCM	14:45135930-45200890
FAS	10:88953813-89029605
FASLG	1:172659103-172666876
FAT1	4:186587794-186726722
FAT4	4:125314918-125492932
FBXO11	2:47789316-47906498
FBXW7	4:152320544-152536092
FGFR1	8:38400215-38468834
FGFR2	10:121478332-121598458
FGFR3	4:1793293-1808872
FH	1:241497511-241519799
FHIT	3:59747277-61251459
FLCN	17:17212212-17237188
FLI1	11:128686535-128813267
FLT3	13:28003274-28100592
FOXO1	13:40555667-40666641
FOXO3	6:108559835-108684774
FOXO4	X:71095851-71103532
FOXP1	3:70954693-71583978
FYB1	5:39105252-39274528
FYN	6:111660332-111873452
G6PC3	17:44070620-44082151
GALE	1:23795599-23800781
GATA1	X:48786540-48794311
GATA2	3:128479427-128493201
GATA3	10:8045378-8075198
GBA1	1:155234452-155244699
GFI1	1:92473043-92486925

GFI1B	9:132944000-132991687
GINS1	20:25391008-25452700
GNA13	17:65009289-65056740
GNAS	20:58839718-58911192
GNB1	1:1785285-1892292
GP1BA	17:4932277-4935023
GP1BB	22:19723539-19724771
GP9	3:129060779-129062406
GPC3	X:133535745-133987100
GREM1	15:32718004-32745106
GRHL2	8:101492439-101669726
GSKIP	14:96363452-96387288
H1-4	6:26156329-26157115
H3C11	6:27871845-27872346
HAX1	1:154272355-154275875
HCLS1	3:121631399-121660927
HLTF	3:149030127-149086554
HNF1A	12:120978543-121002512
HNRNPK	9:83968083-83980631
HOXA11	7:27181157-27185232
HOXB13	17:48724763-48728750
HPS1	10:98410939-98446963
HPS3	3:149129638-149173732
HPS4	22:26443107-26483931
HPS5	11:18278668-18322198
HPS6	10:102065349-102068036
HRAS	11:532242-537321
HVCN1	12:110627841-110704950
ID3	1:23557926-23559501
IDH1	2:208236229-208266074
IDH2	15:90083045-90102477
IFNGR2	21:33403413-33479348

IGF2R	6:159969082-160113507
IGLL5	22:22887780-22896111
IKZF1	7:50304068-50405101
IKZF3	17:39757718-39864312
IL17RA	22:17084954-17115693
IL2RG	X:71107404-71112108
IL7R	5:35852695-35879603
INPP5D	2:233059967-233207903
IRF4	6:391739-411443
IRF8	16:85899116-85922606
ITGA2B	17:44372180-44389649
ITGB3	17:47253827-47313743
ITK	5:157142933-157255185
JAGN1	3:9890574-9894349
JAK1	1:64833223-65067754
JAK2	9:4984390-5129948
JAK3	19:17824780-17848071
KDM6A	X:44873188-45112779
KDR	4:55078481-55125595
KIF23	15:69414246-69448427
KIT	4:54657267-54740783
KITLG	12:88492793-88580851
KLF1	19:12884422-12887201
KLF2	19:16324826-16328685
KLF6	10:3775996-3785281
KLHDC8B	3:49171598-49176486
KLHL6	3:183487551-183555706
KMT2A	11:118436456-118526832
KMT2C	7:152134922-152436644
KMT2D	12:49018975-49060794
KRAS	12:25205246-25250936
LAMTOR2	1:156054782-156058506

LAPTM5	1:30732469-30757774
LCK	1:32251244-32286165
LEF1	4:108047545-108168956
LIG4	13:108207439-108218368
LLGL2	17:75525080-75575209
LMO2	11:33858576-33892076
LRIG3	12:58872149-58920504
LRRC4	7:128027071-128032107
LUC7L2	7:139340359-139423457
LYST	1:235661041-235883724
LZTR1	22:20982269-20999032
MAD2L2	1:11658918-11691811
MAF	16:79585843-79600737
MAFB	20:40685848-40689236
MAGT1	X:77825747-77899271
MAML1	5:179732822-179777283
MAP2K1	15:66386837-66491656
MAP2K2	19:4090321-4124122
MAP2K4	17:12020829-12143830
MAP3K14	17:45263119-45317029
MASTL	10:27154824-27187953
MAX	14:65006174-65102695
MBD4	3:129430947-129440179
MECOM	3:169083499-169663775
MED12	X:71118543-71144103
MEF2B	19:19145567-19192131
MEN1	11:64803510-64811294
MET	7:116672196-116798377
MFHAS1	8:8783354-8893630
MGA	15:41621134-41773081
MITF	3:69739456-69968336
MLH1	3:36993226-37050896

MLH3	14:75013769-75051532
MLLT10	10:21524646-21743630
MN1	22:27748277-27801756
MPL	1:43337818-43354466
MRE11	11:94415570-94493885
MRTFA	22:40410281-40636719
MSH2	2:47403067-47663146
MSH6	2:47695530-47810063
MUC1	1:155185824-155192916
MUTYH	1:45329163-45340893
MYB	6:135181308-135219173
MYC	8:127735434-127742951
MYCN	2:15940550-15947007
MYD88	3:38138552-38143024
MYH9	22:36281280-36388010
MYO5A	15:52307281-52529132
MYSM1	1:58643440-58700090
NAF1	4:163110073-163166890
NAPRT	8:143574785-143578649
NBEAL2	3:46979666-47009704
NBN	8:89924515-90003228
NCOA4	10:46005088-46030623
NCOR1	17:16029065-16218185
NDRG1	8:133237175-133302022
NF1	17:31094927-31382116
NF2	22:29603553-29698598
NFKBIE	6:44258166-44265788
NHP2	5:178149463-178153894
NIPBL	5:36876769-37066413
NKX2-1	14:36516392-36521149
NLRP2	19:54953130-55001142
NOP10	15:34339159-34343180

NOTCH1	9:136494433-136546048
NOTCH2	1:119911553-120100779
NPAT	11:108157215-108222638
NPM1	5:171387116-171411810
NR4A3	9:99821855-99866891
NRAS	1:114704469-114716771
NSD1	5:177131830-177300213
NSD2	4:1871393-1982207
NSUN2	5:6599239-6633291
NT5C2	10:103087185-103277605
NTHL1	16:2039815-2047866
NTRK1	1:156815636-156881850
NTRK3	15:87859751-88256791
NUP98	11:3671083-3797792
NXF1	11:62792123-62806302
PALB2	16:23603160-23641321
PARN	16:14435700-14632728
PAX5	9:36833269-37034268
PBRM1	3:52545352-52685917
PCLO	7:82754012-83162930
PDGFRA	4:54229280-54298245
PDGFRB	5:150113839-150155872
PGM3	6:83147324-83193936
PHF6	X:134373288-134428791
PHOX2B	4:41744082-41748725
PIGA	X:15319452-15335554
PIK3CD	1:9629889-9729114
PIK3R1	5:68215740-68301821
PIM1	6:37170152-37175428
PLCG1	20:41136960-41196801
PLCG2	16:81779279-81962685
PML	15:73994673-74047827

PMS1	2:189784085-189877629
PMS2	7:5970925-6009130
POLD1	19:50384204-50418018
POLE	12:132623753-132687376
POLH	6:43576185-43620523
POT1	7:124822386-124929983
PPM1D	17:60600193-60666280
PRDM1	6:105993463-106109939
PRDM9	5:23443586-23528093
PRF1	10:70597348-70602759
PRKACG	9:69012504-69014113
PRKAR1A	17:68511780-68551319
PRKCB	16:23835983-24220611
PRPF40B	12:49568218-49644666
PRPF8	17:1650629-1684867
PRPS1	X:107628428-107651993
PSMB5	14:23016543-23035230
PTCH1	9:95442980-95517057
PTEN	10:87862638-87971930
PTPN11	12:112418351-112509918
PTPN2	18:12785478-12929643
PTPRD	9:8314246-10613002
PTPRJ	11:47980425-48170839
RAB27A	15:55202966-55319113
RAC2	22:37225270-37259594
RAD21	8:116845934-116874776
RAD50	5:132556019-132646349
RAD51	15:40694774-40732340
RAD51C	17:58692573-58735611
RAD51D	17:35092221-35121522
RAF1	3:12582101-12664201
RAG1	11:36510372-36593156

RASA2	3:141487027-141615344
RB1	13:48303744-48599436
RBBP6	16:24537693-24572863
RBM8A	1:145921556-145927678
RECQL	12:21468910-21501669
RECQL4	8:144511288-144517845
RECQL5	17:75626845-75667189
RELN	7:103471381-103989658
REST	4:56907876-56966808
RFWD3	16:74621399-74666877
RHBDF2	17:76470891-76501790
RHOA	3:49359139-49412998
RIF1	2:151409883-151508013
RIT1	1:155897808-155911404
RMRP	9:35657754-35658017
RNF168	3:196468783-196503768
ROS1	6:117287353-117425942
RPL10	X:154389955-154409168
RPL11	1:23691742-23696835
RPL15	3:23916591-23924374
RPL18	19:48615328-48619184
RPL22	1:6185020-6209389
RPL23	17:38847860-38853764
RPL26	17:8377516-8383213
RPL27	17:42998273-43002959
RPL31	2:101002229-101024032
RPL35	9:124857880-124861981
RPL35A	3:197950190-197956610
RPL36	19:5674947-5691875
RPL5	1:92832013-92841924
RPS10	6:34417454-34426069
RPS15	19:1438358-1440495

RPS17	15:82536750-82540459
RPS19	19:41860255-41872925
RPS20	8:56067254-56074510
RPS24	10:78033760-78056813
RPS26	12:56041351-56044697
RPS27	1:153990762-153992155
RPS27A	2:55231903-55235853
RPS28	19:8321158-8323340
RPS29	14:49570984-49599164
RPS7	2:3575260-3580920
RRAS	19:49635292-49640143
RTEL1	20:63657810-63696253
RUNX1	21:34787801-36004667
SAMD9	7:93099513-93118023
SAMD9L	7:93130056-93148385
SAMHD1	20:36890229-36951893
SBDS	7:66987680-66995693
SBF2	11:9776776-10304877
SDHA	5:218303-257082
SDHAF2	11:61430042-61446839
SDHB	1:17018664-17054151
SDHC	1:161314381-161363206
SDHD	11:112086824-112120016
SEC23B	20:18507520-18561415
SETBP1	18:44680173-45068510
SETD2	3:47016428-47164113
SETDB1	1:150926263-150964744
SF1	11:64764606-64778786
SF3A1	22:30331988-30356919
SF3B1	2:197388515-197435079
SH2B3	12:111405923-111451623
SH2D1A	X:124227868-124373197

SHANK2	11:70467854-71252577
SHOC2	10:110919367-111017307
SLC37A4	11:119023751-119030906
SLFN14	17:35543985-35560819
SLX4	16:3581181-3611606
SMAD4	18:51028528-51085045
SMARCA2	9:1980290-2193624
SMARCA4	19:10960932-11079426
SMARCB1	22:23786931-23838009
SMARCD2	17:63832081-63843065
SMC1A	X:53374149-53422728
SMC3	10:110567684-110606048
SMO	7:129188633-129213545
SOCS1	16:11254417-11256204
SOS1	2:38962206-39124345
SOS2	14:50117130-50231578
SP140	2:230203110-230313215
SPEN	1:15836095-15940456
SPI1	11:47354860-47409369
SPRED1	15:38252836-38357249
SRC	20:37344685-37406050
SRP54	14:34981957-35029686
SRP72	4:56467617-56503681
SRSF2	17:76734115-76737333
STAG2	X:123960212-124422664
STAT3	17:42313324-42388568
STAT5B	17:42199176-42288633
STIM1	11:3854527-4093210
STK11	19:1177558-1228431
STN1	10:103856806-103918332
STX11	6:144150487-144191939
STXBP2	19:7636772-7647873

SUFU	10:102503972-102633535
SUZ12	17:31937007-32001038
SYK	9:90801787-90898549
SYNE1	6:152121687-152637801
TAFAZZIN	X:154411524-154421726
TAL1	1:47216290-47232225
TBL1XR1	3:177019340-177228000
TCF3	19:1609291-1652615
TCF4	18:55222185-55664787
TCF7	5:134114681-134151865
TCF7L2	10:112950247-113167678
TCIRG1	11:68039025-68050895
TERC	3:169764520-169765060
TERF2IP	16:75647773-75761872
TERT	5:1253147-1295068
TET2	4:105145875-105279816
TFE3	X:49028726-49043410
THPO	3:184371935-184381968
TINF2	14:24238286-24242663
TLX1	10:101131300-101137789
TLX3	5:171309248-171312139
TMEM127	2:96248514-96266047
TNFAIP3	6:137867214-137883314
TNFRSF13B	17:16929816-16972118
TNFRSF14	1:2555639-2565382
TOX	8:58805412-59119147
TP53	17:7661779-7687546
TPP1	11:6612768-6619448
TRAF3	14:102777449-102911500
TRIM24	7:138460259-138589996
TSC1	9:132891348-132946874
TSC2	16:2047967-2089491

TSR2	X:54440404-54448032
TUBB1	20:59019429-59026654
TYK2	19:10350533-10380608
U2AF1	21:43092956-43107570
U2AF2	19:55654146-55674716
UBA2	19:34428352-34471251
UBE2T	1:202331544-202341984
UBR5	8:102252273-102412759
UNC13D	17:75827225-75844785
UROS	10:125784980-125823288
USB1	16:57999546-58021618
USP7	16:8892097-8975328
USP9X	X:41085445-41236579
VAV1	19:6772708-6857366
VHL	3:10141778-10153667
VPS13B	8:99013266-99877580
VPS45	1:150067279-150145329
VWF	12:5948877-6124770
WAC	10:28532493-28623112
WAS	X:48676596-48691431
WDR1	4:10068089-10116972
WIF1	12:65050626-65121305
WIPF1	2:174559572-174682916
WRAP53	17:7686071-7703502
WRN	8:31033788-31176138
WT1	11:32387775-32435564
XBP1	22:28794555-28800597
XIAP	X:123859712-123913972
XPA	9:97674909-97697340
XPC	3:14145147-14178621
XPO1	2:61476032-61538741
XRCC2	7:152644776-152676193

ZBTB16	11:114059041-114256765
ZBTB7A	19:4043303-4066899
ZCCHC8	12:122471600-122500932
ZEB2	2:144364364-144521057
ZFP36L2	2:43222402-43226606
ZNF217	20:53567071-53609907
ZNF384	12:6666477-6689572
ZRSR2	X:15790156-15830694

Supplementary Table 3: P/LP variants with moderate evidence for association to hematopoietic malignancy risk.

Gene	Variant Type	Variant cDNA	Variant protein	ACMG/AMP Pathogenicity	ACMG/AMP Codes Applied	Germline Source	Classified in ClinVar	Summary of literature for germline risk
ERBB2	Frameshift	c.30dup	p.(Leu11AlafsTer99)	LP	PM2 supporting, PVS1	BMRS	No	*ERBB2* variant identified to segregate with disease in a family with MPN/Melanoma. Odds ratio of 3.5 for MPN compared to healthy controls[11]. *ERBB2* variants were found in 2 cases with familial cancer (multiple types)[12]. They were also found in breast cancer family[13]. However, all these variants reported are missense and some are reported to stabilize the protein and activate HER2 signaling.
CASP10	Frameshift	c.947-950del	p.(Trp316LeufsTer11)	LP	PVS1, PM2 supporting	BMRS	No	*CASP10* is associated with Autoimmune lymphoproliferative syndrome (ALPS) with autosomal dominant inheritance. ALPS is associated with cancer risk, most strongly with lymphoma[14,15].
DHX34	Stopgain[+]	c.1621C>T	p.(Arg541Ter)	LP	PVS1, PM2 supporting	BMRS	No	*DHX34* is involved in NMD, splicing, and hematopoietic differentiation. It has been previously connected to 4 families with MDS/AML, and functional studies showed that the variant abrogates its activity in NMD[16]. Germline missense and frameshift VUS were also identified in 3.2% of patients with marrow hypocellularity[17]. Additional functional assays show that loss of DHX34 regulates splicing of pre-mRNAs and results in differentiation blockade of erythroid and myeloid lineages[18].
	Frameshift[+]	c.2463_2469del	p.(Ser821ArgfsTer50)	LP	PVS1, PM2 supporting	BMRS	No	
PRF1	Frameshift	c.50del	p.(Leu17ArgfsTer34)	P	PVS1, PS3, PS4	BMRS	Yes	Homozygous loss of *PRF1* leads to familial hemophagocytic lymphohystiocytosis in infants. There is evidence that partial loss of *PRF1* can lead to delayed-onset FHL and hematopoietic malignancies[19]. Another paper found higher incidence of *PRF1* variants than normal controls in patients with Anaplastic large cell lymphoma[20]. Two other case reports documented hemizygous *PRF1* variants in cases of FLH[21,22].
	Frameshift	c.50del	p.(Leu17ArgfsTer34)	P	PVS1, PS3, PS4	BMRS	Yes	
	Frameshift	c.50del	p.(Leu17ArgfsTer34)	P	PVS1, PS3, PS4	BMRS	Yes	
	Missense	c.666C>A	p.(His222Gln)	P	PS4, PS3, PM1, PP3	BMRS	Yes	

FANCA	Frameshift	c.1615del	p.(Asp539ThrfsTer66)	LP	PVS1, PM2 supporting	BMRS	Yes	*FANCA* heterozygous variants are generally not thought to cause disease. A study of 396 Fanconi anemia relatives that were heterozygous carriers did not find increased overall cancer risks, although sample sizes were underpowered for evaluating individual genes[23]. However, one study found *FANCA* variants significantly enriched in aplastic anemia and AML patients[24]. Another study found heterozygous *FANCA* variants at a higher rate in AML than would be expected by chance[25]. Other studies indicate a moderate increase in risk to breast cancer [26–28]
RASA2	Stopgain	c.874C>T	p.(Gln292Ter)	LP	PVS1, PM2 supporting	BMRS	No	*RASA2* loss of function variants were seen in three patients with Noonan syndrome that otherwise lacked a variant in a Noonan syndrome associated gene. Functional studies showed that heterozygous knockdown of *RASA2* resulted in RAS-ERK pathway activation, further supporting a causative role[29]. *RASA2* is mutated somatically in 5% of melanomas and also present in other cancers[30]. In COSMIC, it is mutated somatically in 0.85% of hematopoietic and lymphoid malignancies, further supporting its role as a tumor suppressor gene.

ACMG - American College of Medical Genetics and Genomics, AMP - Association of Molecular Pathology, P - pathogenic, LP - likely pathogenic, AR - autosomal recessive, AD - autosomal dominant. BMRS - Bone marrow remission sample, BMSC - Bone marrow stromal cells. +Variants in the same patient, but not confirmed in trans.

Supplementary Table 4: P/LP variants in genes with limited evidence for association to hematopoietic malignancy risk.

Gene	Variant Type	Variant cDNA	Variant protein	ACMG/AMP Pathogenicity	ACMG/AMP Codes Applied	Germline Source	Classified in ClinVar	Summary of literature for germline risk
DDX54	Splice	c.2196-2A>C	Retention of intron 17/19	LP	PM2 supporting, PVS1	BMRS	No	*DDX54* contributes to genomic stability. GWAS has shown an association between *DDX54* and hematopoietic malignancy[31].
DNAH5	Stopgain	c.328A>T	p.(Lys110Ter)	LP	PVS1, PM2 supporting	BMRS	No	*DNAH5* germline variants were identified in adult AML patients[32]. However, LOF variants are somewhat common in gnomAD (522 individuals). Primary cilia are microtubule-based organelles found on the surface of almost all human bone marrow cells. Primary cilia respond to developmental signaling pathways like Hedgehog (Hh) and Wnt/β-catenin. AML cells show reduced frequency of primary cilia with abnormal morphology[33].
	Stopgain	c.11725C>T	p.(Arg3909Ter)	LP	PVS1, PP4	BMRS	Yes	
	Frameshift	c.13194_13197del	p.(Asp4398GlufsTer16)	P	PVS1, PM3, PP4	BMRS	Yes	
	Stopgain	c.9502C>T	p.(Arg3168Ter)	LP	PVS1, PM2 supporting	BMRS	Yes	
DNAH9	Frameshift	c.308del	p.(Phe103SerfsTer31)	LP	PVS1, PM3 supporting	BMRS	Yes	Similarly to *DNAH5, DNAH9* germline variants were identified in adult AML patients[32]. AML cells show reduced frequency of primary cilia with abnormal morphology[33].
	Stopgain	c.9421C>T	p.(Gln3141Ter)	LP	PVS1, PM2 supporting	BMRS	No	
ATG2B	Splice	c.5579+1G>T	Partial loss of exon 38/42.	LP	PVS1 (RNA), PM2 supporting	BMRS	No	*ATG2B* has been documented to contribute to familial MPNs with a germline duplication. Frameshift mutations in *ATG2B* are common in gastric cancers with high microsatellite instability[34]. *ATG2B* variants are predictive of survival in colorectal cancer[35]. *ATG2B* regulates cancer stemness in TNBC[36].

ACMG - American College of Medical Genetics and Genomics, AMP - Association of Molecular Pathology, P - pathogenic, LP - likely pathogenic, AR - autosomal recessive, AD - autosomal dominant. BMRS - Bone marrow remission sample, BMSC - Bone marrow stromal cells.

Supplementary Table 5: P/LP variants with limited evidence for association to solid tumor risk.

Gene	Variant Type	Variant cDNA	Variant protein	ACMG/AMP Pathogenicity	ACMG/AMP Codes Applied	Germline Source	Classified in ClinVar	Summary of literature for germline risk
SMO	Missense	c.1921C>G	p.(Pro641Ala)	LP	PS3, PP1, PP3	BMRS	Yes	Germline *SMO* P641A was found in a patient with non-small cell lung cancer, and his daughter had the variant and developed a skin basal cell carcinoma at a young age. Functional evidence showed activation of hedgehog pathway genes that was similar to overexpression of *SMO*[37].
	Missense	c.1921C>G	p.(Pro641Ala)	LP	PS3, PP1, PP3	BMRS	Yes	
ROS1	Frameshift	c.350del	p.(Leu117Tyrfs Ter11)	LP	PVS1, PM2 supporting	BMRS	No	*ROS1* germline variants were identified in two families with *BRCA1/2* negative breast cancer[38].
	Stopgain	c.4190G>A	p.(Trp1397Ter)	LP	PVS1, PM2 supporting	BMRS	No	
SLX4	Frameshift	c.3895_389 6del	p.(Arg1299Glyf sTer35)	LP	PVS1, PM2 supporting	BMSC	Yes	*SLX4* heterozygous variants are generally not thought to cause disease, although some publications show that *SLX4* heterozygous loss of function variants, although rare, have been found in cases of familial breast cancer [39,40]
	Stopgain	c.928C>T	p.(Arg310Ter)	LP	PVS1, PM2 supporting	BMRS	No	
FANCM	Stopgain	c.5791C>T	p.(Arg1931Ter)	LP	PVS1 strong, PS3-supportin g, PM1	BMRS	Yes	A large-scale analysis of >2000 familial BC without *BRCA1/2* found an increased OR for *FANCM* (OR, 2.44; 95% CI, 1.08-5.59; P = .02)[41]. This variant Arg1931Ter associates with ER-negative breast cancer risk (OR = 1.96; P = 0.006). The variant affected cell survival and chromosomal stability[42].
RECQL5	Stopgain	c.2791A>T	p.(Lys931Ter)	LP	PVS1 (RNA), PM2 supporting	BMRS	No	*RECQL5* is involved in homologous recombination. It has been proposed to be involved in breast cancer susceptibility and was found in familial cases without *BRCA1/2*[43]. Another study found an association with *RECQL5* polymorphisms in a Chinese population of breast cancer patients[44].

ACMG - American College of Medical Genetics and Genomics, AMP - Association of Molecular Pathology, P - pathogenic, LP - likely pathogenic, AR - autosomal recessive, AD - autosomal dominant. BMRS - Bone marrow remission sample, BMSC - Bone marrow stromal cells.

Supplementary Table 6: Heterozygous P/LP variants in genes with autosomal recessive hematopoietic malignancy risk inheritance.

Gene	Variant Type	Variant cDNA	Variant protein	ACMG/AMP Pathogenicity	ACMG/AMP Codes Applied	Germline Source	Classified in ClinVar	Summary of literature for germline risk
CTC1	Frameshift	c.1360del	p.(Glu454SerfsTer9)	LP	PVS1, PP4	BMSC	Yes	Heterozygous *CTC1* variants may contribute to the onset of acquired BMF and clonal outgrowth of PNH clones. Heterozygous *CTC1 is* enriched in AA/PNH cases and associated with shortened telomeres (mean 7 as compared to mean 15 in age-matched controls). However, AA/PNH patients without telomere variants also had shortened telomeres[45].
	Stopgain	c.277C>T	p.(Gln93Ter)	LP	PVS1, PM2 supporting	BMRS	Yes	
FANCD2	Stopgain	c.982C>T	p.(Arg328Ter)	LP	PVS1, PM2 supporting	BMRS	Yes	There is no published evidence of association of heterozygous *FANCD2* to familial cancer.
RAD50	Stopgain	c.832C>T	p.(Arg278Ter)	LP	PVS1, PP4	BMRS	Yes	One study found *RAD50* variants are not associated with increased breast cancer risk, but with worse survival[46]. A large meta-analysis showed no increased risk of breast cancer or other cancers for *RAD50*[47].
RPS27A	Splice	c.-17-98T>A	Retention of intron 1 (upstream of start)	LP	PVS1 (RNA), PM2 supporting	BMRS	No	There is no published evidence of heterozygous *RPS27A* variants in familial cancer.
STXBP2	Missense	c.1654G>A	p.(Gly552Ser)	LP	PS3, PM3, PP3	BMRS	Yes	There is no published evidence of heterozygous *STXBP2* variants in familial cancer.
VPS45	Frameshift	c.695_696del	p.(Tyr232SerfsTer16)	LP	PVS1, PM2 supporting	BMRS	Yes	There is no published evidence of heterozygous *VPS45* variants in familial cancer.
XRCC2	Frameshift	c.96del	p.(Phe32LeufsTer30)	LP	PVS1, PM2 supporting	BMSC	Yes	*XRCC2* c.96delT is a founder variant in Poland. One study did not find an association to breast cancer[48].

ACMG - American College of Medical Genetics and Genomics, AMP - Association of Molecular Pathology, P - pathogenic, LP - likely pathogenic, AR - autosomal recessive, AD - autosomal dominant. BMRS - Bone marrow remission sample, BMSC - Bone marrow stromal cells.

Supplementary Table 7: Heterozygous P/LP variants in genes with autosomal recessive solid tumor risk inheritance.

Gene	Variant Type	Variant cDNA	Variant protein	ACMG/AMP Pathogenicity	ACMG/AMP Codes Applied	Germline Source	Classified in ClinVar	Summary of literature for germline risk
MUTYH	Missense	c.1187G>A	p.(Gly396Asp)	P	PS3, PS4, PP3	BMRS	Yes	Heterozygous variants in *MUTYH* are associated with a slightly increased risk of colorectal cancer[49]. Another large study found the following hazard ratios and 95% CIs for heterozygous *MUTYH* carriers: gastric cancer 9.3 (6.7-13); hepatobiliary cancer 4.5 (2.7-7.5); endometrial cancer 2.1 (1.1-3.9) and breast cancer 1.4 (1.0-2.0)[50]. Other studies also found elevated risk of liver and gastric cancers and a slightly increased risk of breast cancer for heterozygous *MUTYH* [51,52]. Another study confirmed a higher frequency of heterozygous *MUTYH* variants in individuals with cancer and attributed this to loss of heterozygosity of the functional allele[53].
	Missense	c.1187G>A	p.(Gly396Asp)	P	PS3, PS4, PP3	BMRS	Yes	
	Missense	c.1187G>A	p.(Gly396Asp)	P	PS3, PS4, PP3	BMRS	Yes	
	Missense	c.1187G>A	p.(Gly396Asp)	P	PS3, PS4, PP3	BMRS	Yes	
	Missense	c.536A>G	p.(Tyr179Cys)	P	PS3, PS4, PP3	BMRS	Yes	
NTHL1	Stopgain	c.835C>T	p.(Gln279Ter)	P	PVS1 strong, PM3 supporting, PS3	BMRS	Yes	There is insufficient evidence of *NTHL1* heterozygous carriers having an increased risk of cancer, although one study found a low increase in breast cancer risk[54].

RECQL4	Frameshift	c.3072del	p.(Val1026Cy sfsTer18)	P	PVS1, PM2 supporting, PM3 supporting	BMRS	Yes	Homozygous variants in *RECQL4* cause Rothmund-Thomson syndrome, associated with increased risk of childhood cancer. Heterozygous loss of function variants in RECQL4 were enriched in pediatric osteosarcoma patients (odds ratio=7.1, 95% CI, 2.9-17)[55]. However, a study of 123 heterozygous *RECQL4* carriers did not show an increased incidence in cancer[56].
XPC	Stopgain	c.463C>T	p.(Arg155Ter)	LP	PVS1, PM2 supporting	BMRS	Yes	*XPC* is known to confer cancer risk in homozygous form. One study showed that *XPC* polymorphism carriers have a slight increase in risk for breast, bladder, head and neck, and lung cancer[57]. A study of lung adenocarcinoma in mice showed a dose-dependent effect, where mice heterozygous for *XPC* developed an intermediate number of tumors when exposed to cigarette smoke[58]. *XPC* polymorphisms associated with survival in AML patients[59].

ACMG - American College of Medical Genetics and Genomics, AMP - Association of Molecular Pathology, P - pathogenic, LP - likely pathogenic, AR - autosomal recessive, AD - autosomal dominant. BMRS - Bone marrow remission sample, BMSC - Bone marrow stromal cells.

Supplementary Table 8: Heterozygous P/LP variants in genes associated with other hematopoietic diseases or developmental disorders with autosomal recessive inheritance.

Gene	Variant Type	Variant cDNA	Variant protein	ACMG/AMP Pathogenicity	ACMG/AMP Codes Applied	Germline Source	Classified in ClinVar	Summary of literature for germline risk
ADAMTS13	Missense	c.559G>C	p.(Asp187His)	LP	PS3 moderate, PM1, PM3, PP3	BMRS	Yes	Homozygous or compound heterozygous variants are associated with Thrombotic thrombocytopenic purpura, a bleeding disorder. Carriers of *ADAMTS13* are generally considered unaffected. However, one study did find decreased levels of *ADAMTS13* expression in ALL patients, and lower expression associated with patients with infections and high-risk status[60]. Low *ADAMTS13* levels were also found in AML patients and correlated with worse BMT outcomes[61].
GBA1	Missense	c.1483G>C	p.(Ala495Pro)	P	PS3, PS4, PM3	BMRS	Yes	There is moderate evidence for homozygous *GBA1* variants in hematopoietic malignancy risk[62], but heterozygous *GBA1* variants are not documented to increase cancer risk. However, one study found decreased *GBA1* expression in liver cancer patients, which associated with vascular invasion and advanced stage disease due to activation of the WNT signaling pathway[63].
	Missense	c.882T>G	p.(His294Gln)	LP	PM3 Strong, PS4 Moderate	BMRS	Yes	
	Missense	c.1226A>G	p.(Asn409Ser)	LP	PS3 moderate, PS4, PM1, PM3, PP1, PP2	BMRS	Yes	
VPS13B	Frameshift	c.5120_5124 del	p.(Lys1707Serfs Ter9)	LP	PVS1, PM2 supporting	BMRS	No	Homozygous *VPS13B* variants can cause Cohen syndrome, which involves neutropenia. There is no published evidence of heterozygous carriers being affected.

ACMG - American College of Medical Genetics and Genomics, AMP - Association of Molecular Pathology, P - pathogenic, LP - likely pathogenic, AR - autosomal recessive, AD - autosomal dominant. BMRS - Bone marrow remission sample, BMSC - Bone marrow stromal cells.

Supplementary Table 9: P/LP variants in genes with a known somatic role in AML but no prior evidence for germline risk to malignancy.

Gene	Variant Type	Variant cDNA	Variant protein	ACMG/AMP Pathogenicity	ACMG/AMP Codes Applied	Germline Source	Classified in ClinVar	Summary of literature for germline risk
ARHGEF12	Stopgain	c.76C>T	p.(Arg26Ter)	LP	PVS1, PM2 supporting	BMRS	No	*ARHGEF12* has a known somatic role in leukemia. Heterozygous loss of function variants are rare in GnomAD (20 individuals).
CUX1	Frameshift	c.1754del	p.(Phe585SerfsTer11)	LP	PVS1, PM2 supporting	BMRS	Yes	*CUX1* somatic variants are known to contribute to myeloid malignancy[64]. *CUX1* heterozygous germline variants cause mild to moderate intellectual disability/developmental delay of variable phenotype that resolves in adulthood[65]. Heterozygous loss of function variants are rare in GnomAD (11 individuals).
PRPF8	Frameshift	c.605_606del	p.(Pro202ArgfsTer7)	LP	PVS1, PM2 supporting	BMRS	No	*PRPF8* has a known somatic mechanism for AML and MDS. Its inheritance is AD for Retinitis pigmentosa, but average age of onset is 35. Heterozygous loss of function variants are rare in GnomAD (29 individuals).
	Frameshift	c.6991del	p.(Glu2331ArgfsTer28)	LP	PVS1 strong, PM2 supporting, PP1	BMRS	Yes	
BRCC3	Splice	c.196-2del	Retention of intron 3/10.	LP	PVS1 (RNA), PM2 supporting, PP3	BMRS	No	*BRCC3* is a component of the DNA repair pathway. Loss of function variants are rare in GnomAD (4 heterozyotes, 2 homozygotes). Somatic mutations have been reported in MDS and MPNs[66].
BRD4	Splice	c.3782+2T>G	Loss of exon 18/20	LP	PS3 (RNA), PM2 supporting, PP3	BMSC	No	*BRD4* is well-known for somatic mutations in cancer. Heterozygous LOF variants cause pre- and postnatal growth defects [67]. Heterozygous loss of function variants are rare in GnomAD (2 individuals).
FGFR3	Splice	c.445+2T>G	Retention of intron 4/17	LP	PVS1 (RNA), PM2 supporting	BMRS	No	*FGFR3* is mostly known for its somatic role. However it's been observed to accumulate in the male germline via clonal expansion in the testes and correlating with age[68]. Germline variants are associated with skeletal disorders. Heterozygous loss of function variants are rare in GnomAD (36 individuals).
INPP5D	Splice	c.349+2T>G	Retention of intron 3/26	LP	PVS1 (RNA), PP3	BMRS	No	*INPP5D* is known for its somatic role in myeloid malignancy. Mouse studies show complete knockout of *INPP5D* is early postnatal lethal due to overproliferation of myeloid cells[69]. The effects of heterozygous loss of function are unknown, although present in 50 GnomAD subjects.

MYO5A	Stopgain	c.2416C>T	p.(Arg806Ter)	LP	PVS1, PM2 supporting	BMRS	No	*MYO5A* is associated with Griscelli syndrome with autosomal recessive inheritance. It has been reported as a fusion partner in pediatric low-grade glioma[70] and upregulation has been seen in melanoma and other cancer types[71]. Heterozygous loss of function variants are rare in GnomAD (36 individuals).
NFKBIE	Frameshift	c.137del	p.(Ile46ThrfsTer3)	LP	PVS1, PM2 supporting	BMRS	No	*NFKBIE* is commonly mutated somatically in B-cell malignancies and activates the NF-κB pathway[72]. Heterozygous loss of function variants are rare in GnomAD (1 individual).
SETDB1	Stopgain	c.1214C>A	p.(Ser405Ter)	LP	PVS1, PM2 supporting	BMSC	No	*SETDB1* has a known somatic role in cancer[73]. Heterozygous loss of function variants are rare in GnomAD (12 individuals).
SYNE1	Stopgain	c.23309T>A	p.(Leu7770Ter)	LP	PVS1, PM2 supporting	BMRS	No	*SYNE1* is commonly mutated somatically in cancer. *SYNE1* is associated with muscular dystrophy (AD) and spinocerebellar ataxia (AR).

ACMG - American College of Medical Genetics and Genomics, AMP - Association of Molecular Pathology, P - pathogenic, LP - likely pathogenic, AR - autosomal recessive, AD - autosomal dominant. BMRS - Bone marrow remission sample, BMSC - Bone marrow stromal cells.

Supplementary Table 10: Patients with multiple P/LP variants.

**USI**	**Genes with P/LP variant**
PATKWH	*BRD4, TRIM24*
PAUSBP	*CHEK2, DDX54*
PAUVWV	*MRE11A, MUTYH*
PAVCNG	*DNAH9, ROS1*
PAWPKR	*HCLS1, MITF*
PAWSJC	*DHX34, DHX34*
PAWUAE	*MUTYH, TNFRSF13B*
PAXELP	*DNAH5, FANCM, PRF1*
PAXJZX	*SBDS, SBDS, TP53*

Supplementary Table 11: Additional information for P/LP variants.

USI	Gene	Variant cDNA	Variant Protein	Variant Type	Transcript	ACMG Pathogenicity	ACMG Criteria	Tissue source	Fibroblast VAF	Remission VAF	Diagnosis VAF	Relapse VAF	RNA VAF	Sex	Age Group	Major Fusion or Somatic Driver	Fusion or Somatic Driver VAF in Remission WGS	MRD (%) at induction 1	FAB	EFS event type ID
PAXLWH	ADAMTS13	c.559G>C	p.(Asp187His)	Missense	NM_139025.5	LP	PS3 moderate, PM1, PM3, PP3	BMRS	NA	0.61	NA	0.31	0.33	M	Child	NUP98-NSD1	0 (FLT3-ITD)	0	M5	Relapse
PAWZAU	ARHGEF12	c.76C>T	p.(Arg26Ter)	Stopgain	NM_015313.3	LP	PVS1, PM2 supporting	BMRS	NA	0.52	0.38	0.61	0.51	F	Child	CBFB-MYH11	0	0	Unknown	Relapse
PAWVCG	ATG2B	c.5579+1G>T	Partial loss of exon 38/42.	Splice	NM_018036.7	LP	PVS1 (RNA), PM2 supporting	BMRS	NA	0.44	0.38	0.53	0*	M	Infant	KMT2A-MLLT3	0	0	Unknown	Relapse
PAWWCW	ATM	c.8395_8404del	p.(Phe2799LysfsTer4)	Frameshift	NM_000051.4	P	PVS1, PM2 supporting, PM3	BMRS	NA	0.37	0.21	0.41	0.32	M	Infant	KMT2A_MLLT10	0	0	M5	Relapse
PAUWFM	BRCA2	c.8377G>A	p.(Gly2793Arg)	Missense	NM-000059.4	P	PS3, PS4, PM2-supporting, PP3	BMRS	NA	0.43	0.49	NA	0.32	M	Infant	CBFA2T3-GLIS2	+21 in tumor WGS and absent in remission WGS	0.9	M7	Relapse
PAUXKI	BRCA2	c.5410-5411del	p.(Val1804LysfsTer2)	Frameshift	NM-000059.4	LP	PVS1, PM2-supporting	BMRS	NA	0.49	0.5	0.4	0.27	M	AYA	RUNX1-RUNX1T1	0	0	Unknown	Relapse
PAVHWK	BRCA2	c.5946del	p.(Ser1982ArgfsTer22)	Frameshift	NM-000059.4	P	PVS1, PS3	BMRS	NA	0.56	0.41	NA	0.32	F	Child	CBFB-MYH11	0	0	Unknown	Censored
PAVKAU	BRCA2	c.5614A>T	p.(Lys1872Ter)	Stopgain	NM-000059.4	P	PVS1, PS4, PM2-supporting	BMRS	NA	0.58	0.42	0.6	NA	F	Infant	KMT2A-MLLT3	0	0	Unknown	Relapse
PAVACZ	BRCC3	c.196-2del	Retention of intron 3/10.	Splice	NM_024332.4	LP	PVS1 (RNA), PM2 supporting, PP3	BMRS	NA	0.45	0.48	NA	0.4	F	Child	KMT2A-MLLT3	0	0	Unknown	Relapse
PATKWH	BRD4	c.3782+2T>G	Loss of exon 18/20	Splice	NM_001379291.1	LP	PS3 (RNA), PM2 supporting, PP3	BMSC	0.3	NA	NA	NA	0*	M	AYA	None	NA	NA	M6	Relapse

PAUYSN	BRIP1	c.2990-2993del	p.(Thr997Argfs Ter61)	Frameshift	NM-032043.3	P	PVS1, PS4	BMRS	NA	0.45	0.43	NA	0*	M	Infant	None	Unknown	0	M5	Censored
PAVDZK	BRIP1	c.139C>G	p.(Pro47Ala)	Missense	NM-032043.3	LP	PS3 moderate, PS4 moderate, PM1, PP3	BMRS	NA	0.48	0.91	1	1.00	M	AYA	MED14-HOXA9 (promoter hijacking)	0	0	Unknown	Relapse
PAVEAF	CASP10	c.947-950del	p.(Trp316Leufs Ter11)	Frameshift	NM-032977.4	LP	PVS1, PM2 supporting	BMRS	NA	0.58	0.32	NA	0.36	M	Child	NUP98-NSD1	0	7.4	Unknown	Relapse
PARNAW	CDKN2A	c.194T>C	p.(Leu65Pro)	Missense	ENST00000304494.10	LP	PS4, PP1 moderate, BP4	BMSC	0.45	NA	NA	NA	0.1	F	Infant	None	NA	NA	M7	Induction failure
PASFHK	CDKN2A	c.194T>C	p.(Leu65Pro)	Missense	ENST00000304494.10	LP	PS4, PP1 moderate, BP4	BMSC	0.32	NA	NA	NA	0.13	M	Child	None	NA	NA	M4	Induction failure
PATAIJ	CDKN2A	c.194T>C	p.(Leu65Pro)	Missense	ENST00000304494.10	LP	PS4, PP1 moderate, BP4	BMSC	0.33	NA	NA	NA	0.07	F	Infant	KMT2A-MLLT10	NA	NA	M5	Induction failure
PAUSBP	CHEK2	c.1100del	p.(Thr410Metfs Ter15)	Frameshift	NM_007194.4	P	PVS1, PS3 supporting, PS4	BMRS	NA	0.63	NA	0.43	0.30	M	Child	KMT2A-AFDN	17p deletion in tumor WGS and absent in remission WGS	0	M5	Relapse
PAUVGD	CHEK2	c.599T>C	p.(Ile157Thr)	Missense	NM_007194.4	LP	PS3, PS4 moderate, PM1, BS1	BMRS	NA	0.64	0.6	0.58	0.42	M	Child	NUP98-NSD1	0	12	M0	Relapse
PAWHML	CHEK2	c.599T>C	p.(Ile157Thr)	Missense	NM_007194.4	LP	PS3, PS4 moderate, PM1, BS1	BMRS	NA	0.54	NA	0.62	0.54	M	AYA	CEBPA	0	0	M2	Relapse
PAWHTL	CHEK2	c.836T>C	p.(Ile157Thr)	Missense	NM_007194.4	LP	PS3 supporting, PS4, PM1	BMRS	NA	0.5	0.48	0.44	NA	M	Child	CBFB-MYH11	0	0.02	M1	Relapse
PAXJGA	CHEK2	c.599T>C	p.(Ile157Thr)	Missense	NM_007194.4	LP	PS3, PS4 moderate, PM1, BS1	BMRS	NA	0.63	0.46	0.46	0.58	F	Child	CBFB-MYH11	0	0	Unknown	Relapse
PATJMY	CTC1	c.1360del	p.(Glu454Serfs Ter9)	Frameshift	NM_025099.6	LP	PVS1, PP4	BMSC	0.53	NA	NA	NA	0.28	M	Child	NUP98-NSD1	NA	NA	M2	Induction failure

PAVIGB	CTC1	c.277C>T	p.(Gln93Ter)	Stopgain	NM_025099.6	LP	PVS1, PM2 supporting	BMRS	NA	0.45	0.55	NA	0.59	F	Infant	KMT2A-MLLT3	0	0	Unknown	Relapse
PAWTWW	CUX1	c.1754del	p.(Phe585SerfsTer11)	Frameshift	NM_001913.5	LP	PVS1, PM2 supporting	BMRS	NA	0.44	0.58	0.47	0.02	M	AYA	KMT2A-ELL	0	0	M5	Relapse
PAUSBP	DDX54	c.2196-2A>C	Retention of intron 17/19	Splice	NM_001111322.2	LP	PM2 supporting, PVS1	BMRS	NA	0.46	NA	0.49	0.9	M	Child	KMT2A-AFDN	0	0	M5	Relapse
PAWSJC	DHX34	c.1621C>T	p.(Arg541Ter)	Stopgain	NM_014681.6	LP	PVS1, PM2 supporting	BMRS	NA	0.66	0.45	0.41	0.48	F	AYA	CBFB-MYH11	0	0	Unknown	Relapse
PAWSJC	DHX34	c.2463_2469del	p.(Ser821ArgfsTer50)	Frameshift	NM_014681.6	LP	PVS1, PM2 supporting	BMRS	NA	0.38	0.32	0.36	0.15	F	AYA	CBFB-MYH11	0	0	Unknown	Relapse
PAUVBS	DNAH5	c.9502C>T	p.(Arg3168Ter)	Stopgain	NM_001369.3	LP	PVS1, PM2 supporting	BMRS	NA	0.39	0.53	NA	0*	F	Child	KMT2A-MLLT1	0.03	4.7	M1	Relapse
PAVDRG	DNAH5	:c.328A>T	p.(Lys110Ter)	Stopgain	NM_001369.3	LP	PVS1, PM2 supporting	BMRS	NA	0.5	0.3	NA	0*	F	Child	DEK_NUP214	0.03	0	Unknown	Censored
PAWBSJ	DNAH5	c.11725C>T	p.(Arg3909Ter)	Stopgain	NM_001369.3	LP	PVS1, PP4	BMRS	NA	0.52	0.34	0.58	0*	M	Child	RUNX1-RUNX1T1	0.04	0	Unknown	Relapse
PAXELP	DNAH5	c.13194_13197del	p.(Asp4398GlufsTer16)	Frameshift	NM_001369.3	P	PVS1, PM3, PP4	BMRS	NA	0.57	0.57	0.45	0*	M	AYA	RUNX1-RUNX1T1	0.04	1.4	Unknown	Relapse
PAVCNG	DNAH9	c.9421C>T	p.(Gln3141Ter)	Stopgain	NM_001372.4	LP	PVS1, PM2 supporting	BMRS	NA	0.45	0.44	NA	0*	M	AYA	CEBPA	0	0	M2	Relapse
PAVCNU	DNAH9	c.308del	p.(Phe103SerfsTer31)	Frameshift	NM_001372.4	LP	PVS1, PM3 supporting	BMRS	NA	0.48	0.44	NA	0*	F	Child	NUP98-HOXA13	0	0	M2	Death
PARBTV	DNMT3A	c.2645G>A	p.(Arg882His)	Missense	NM_022552.5	P	PS4 very strong, PS3, PP3	BMSC	0.54	NA	NA	NA	0.49	M	AYA	None	NA	NA	M1	Induction failure
PAWMKP	ERBB2	c.30dup	p.(Leu11AlafsTer99)	Frameshift	NM_004448.4	LP	PM2 supporting, PVS1	BMRS	NA	0.59	NA	0.44	0*	M	Child	RUNX1-RUNX1T1	0	0	Unknown	Relapse
PAWAMB	FANCA	c.1615del	p.(Asp539ThrfsTer66)	Frameshift	NM_000135.4	LP	PVS1, PM2 supporting	BMRS	NA	0.62	0.44	0.49	0.34	F	Infant	NUP98-NSD1	0	0	Unknown	Relapse

PAVBUX	FANCD2	c.982C>T	p.(Arg328Ter)	Stopgain	NM_033084.6	LP	PVS1, PM2 supporting	BMRS	NA	0.46	0.41	NA	0.38	M	AYA	KMT2A PTD	KMT2A PTD in tumor WGS and absent in remission WGS	0	M4	Relapse
PAXELP	FANCM	c.5791C>T	p.(Arg1931Ter)	Stopgain	NM_020937.4	LP	PVS1 strong, PS3-supporting, PM1	BMRS	NA	0.34	0.43	0.51	0*	M	AYA	RUNX1-RUNX1 T1	0.04	1.4	Unknow n	Relapse
PAUUKZ	FGFR3	c.445+2T>G	Retention of intron 4/17	Splice	NM_000142.5	LP	PVS1 (RNA), PM2 supporting	BMRS	NA	0.6	0.59	0.48	NA	M	Infant	KMT2A-MLLT3	0	0	Unknow n	Relapse
PAUWKY	GATA2	c.241G>T	p.(Gly81Ter)	Stopgain	NM-032638.5	LP	PVS1, PM2 supporting	BMRS	NA	0.43	0.61	NA	0.70	M	Child	None	0	NA	Unknow n	Death
PAVEAF	GBA1	c.1483G>C	p.(Ala495Pro)	Missense	NM_001005742. 3	P	PS3, PS4, PM3	BMRS	NA	0.56	0.22	NA	0*	M	Child	NUP98-NSD1	0	7.4	Unknow n	Relapse
PAVGKS	GBA1	c.1226A>G	p.(Asn409Ser)	Missense	NM_001005742. 3	LP	PS3 moderate, PS4, PM1, PM3, PP1, PP2	BMRS	NA	0.47	0.43	0.56	0*	F	AYA	KMT2A-ELL	0 (FLT3-ITD)	0	NOS	Relapse
PAVKKI	GBA1	c.882T>G	p.(His294Gln)	Missense	NM_001005742. 3	LP	PM3 Strong, PS4 Moderate	BMRS	NA	0.5	0.24	NA	0*	F	Child	NUP98-NSD1	0.04	18	M0	Relapse
PAWPKR	HCLS1	c.1191T>G	p.(Tyr397Ter)	Stopgain	NM_005335.6	LP	PVS1, PM2 supporting	BMRS	NA	0.58	0.72	0.68	NA	F	Child	KMT2A-MLLT3	0	0	Unknow n	Relapse
PAVRJP	INPP5D	c.349+2T>G	Retention of intron 3/26	Splice	NM_005541.5	LP	PVS1 (RNA), PP3	BMRS	NA	0.33	NA	0.33	0.75	M	AYA	KMT2A-MLLT10	0	0	M5	Relapse
PAUMCV	MITF	c.1273G>A	p.(Glu425Lys)	Missense	NM_001354604. 2	LP	PS3 moderate, PS4 moderate, PM1, PP1, BP4	BMRS	NA	0.47	0.54	NA	NA	F	Infant	None	del(13q) present in tumor WGS and absent in remission WGS	0	M6	Censored
PAVMEH	MITF	c.1273G>A	p.(Glu425Lys)	Missense	NM_001354604. 2	LP	PS3 moderate, PS4 moderate, PM1, PP1, BP4	BMRS	NA	0.38	0.21	0.54	0*	F	Child	CEBPA	0	0	Unknow n	Censored

PAWPKR	MITF	c.1273G>A	p.(Glu425Lys)	Missense	NM_001354604.2	LP	PS3 moderate, PS4 moderate, PM1, PP1, BP4	BMRS	NA	0.59	0.44	0.63	NA	F	Child	KMT2A-MLLT3	0	0	Unknown	Relapse
PAUVWV	MRE11	c.1714C>T	p.(Arg572Ter)	Stopgain	NM_005591.4	P	PVS1, PS4	BMRS	NA	0.58	0.4	NA	0*	F	Infant	KMT2A-MYO1F	0	0	Unknown	Censored
PAUMGZ	MUTYH	c.1187G>A	p.(Gly396Asp)	Missense	NM_001128425.2	P	PS3, PS4, PP3	BMRS	NA	0.6	0.53	NA	0*	F	Child	NPM1	0	0	M1	Censored
PAUMUZ	MUTYH	c.1187G>A	p.(Gly396Asp)	Missense	NM_001128425.2	P	PS3, PS4, PP3	BMRS	NA	0.49	0.25	0.41	0.00	F	Child	MYB_CLINT1	0	0	Unknown	Relapse
PAUNVK	MUTYH	c.1187G>A	p.(Gly396Asp)	Missense	NM_001128425.2	P	PS3, PS4, PP3	BMRS	NA	0.5	0.64	NA	0.5	M	Child	RUNX1-RUNX1T1	0	0	Unknown	Censored
PAUVWV	MUTYH	c.536A>G	p.(Tyr179Cys)	Missense	NM_001128425.2	P	PS3, PS4, PP3	BMRS	NA	0.58	0.52	NA	0.63	F	Infant	KMT2A-MYO1F	0	0	Unknown	Censored
PAWUAE	MUTYH	c.1187G>A	p.(Gly396Asp)	Missense	NM_001128425.2	P	PS3, PS4, PP3	BMRS	NA	0.54	0.49	0.43	0.52	M	Infant	KMT2A-MLLT10	0	0	Unknown	Relapse
PAVHLI	MYO5A	c.2416C>T	p.(Arg806Ter)	Stopgain	NM_000259.3	LP	PVS1, PM2 supporting	BMRS	NA	0.61	NA	0.4	NA	F	AYA	CBFB-MYH11	0	0	Unknown	Relapse
PAVBYN	NFKBIE	c.137del	p.(Ile46ThrfsTer3)	Frameshift	ENST00000275015.5	LP	PVS1, PM2 supporting	BMRS	NA	0.5	NA	0.48	0*	F	AYA	CEBPA	0	0	Unknown	Relapse
PAULCA	NTHL1	c.835C>T	p.(Gln279Ter)	Stopgain	NM_002528.7	P	PVS1 strong, PM3 supporting, PS3	BMRS	NA	0.56	0.63	NA	0.45	F	Infant	None	Unable to identify somatic driver	8.9	M6	Death without remission
PAXETC	PALB2	c.3113G>A	p.(Trp1038Ter)	Stopgain	NM_024675.4	P	PVS1, PS4 moderate, PP1	BMRS	NA	0.39	0.42	0.36	0.11	M	AYA	KMT2A_MYOCD	+8 in tumor WGS and absent in remission WGS	37	M5	Relapse
PAUNSV	PRF1	c.50del	p.(Leu17ArgfsTer34)	Frameshift	NM_005041.6	P	PVS1, PS3, PS4	BMRS	NA	0.36	NA	0.38	0.12	M	Child	CEBPA	0	0	Unknown	Relapse
PAVFDW	PRF1	c.50del	p.(Leu17ArgfsTer34)	Frameshift	NM_005041.6	P	PVS1, PS3, PS4	BMRS	NA	0.49	0.43	NA	0.75	F	AYA	NPM1	0	0	M2	Relapse

PAVHCV	PRF1	c.50del	p.(Leu17ArgfsTer34)	Frameshift	NM_005041.6	P	PVS1, PS3, PS4	BMRS	NA	0.53	0.61	NA	0.12	M	Child	CBFB-MYH11	0	0	Unknown	Censored
PAXELP	PRF1	c.666C>A	p.(His222Gln)	Missense	NM_005041.6	P	PS4, PS3, PM1, PP3	BMRS	NA	0.5	0.67	0.55	0.67	M	AYA	RUNX1-RUNX1T1	0.04	1.4	Unknown	Relapse
PAUZVP	PRPF8	c.605_606del	p.(Pro202ArgfsTer7)	Frameshift	NM_006445.4	LP	PVS1, PM2 supporting	BMRS	NA	0.5	0.55	0.38	0.22	M	AYA	NUP214-ABL1	XXYY present in tumor WGS and absent in remission WGS	5	Unknown	Relapse
PAXMLI	PRPF8	c.6991del	p.(Glu2331ArgfsTer28)	Frameshift	NM_006445.4	LP	PVS1 strong, PM2 supporting, PP1	BMRS	NA	0.39	NA	0.44	0.44	M	AYA	KMT2A-MLLT10	0	0	M5	Relapse
PAXBAZ	RAD50	c.832C>T	p.(Arg278Ter)	Stopgain	NM_005732.4	LP	PVS1, PP4	BMRS	NA	0.45	0.58	0.63	0.56	F	Child	TEC-MLLT10	0	0	M7	Relapse
PAVTDU	RAD51C	c.577C>T	p.(Arg193Ter)	Stopgain	NM-058216.3	P	PVS1, PS4	BMRS	NA	0.63	NA	0.51	0*	F	Child	None	0	0	M2	Relapse
PAVLJH	RASA2	c.874C>T	p.(Gln292Ter)	Stopgain	NM_006506.5	LP	PVS1, PM2 supporting	BMRS	NA	0.43	0.43	0.5	0.23	F	AYA	CBFB-MYH11	0	0	Unknown	Relapse
PAWCJK	RECQL4	c.3072del	p.(Val1026CysfsTer18)	Frameshift	NM_004260.4	P	PVS1, PM2 supporting, PM3 supporting	BMRS	NA	0.61	0.33	0.49	0.29	F	AYA	NPM1	0	0	Unknown	Relapse
PAUUKK	RECQL5	c.2791A>T	p.(Lys931Ter)	Stopgain	NM_004259.7	LP	PVS1 (RNA), PM2 supporting	BMRS	NA	0.38	0.62	NA	0.43	F	Infant	KAT6A-CREBBP	0	0	M5	Relapse
PAVCNG	ROS1	c.4190G>A	p.(Trp1397Ter)	Stopgain	NM_002944.3	LP	PVS1, PM2 supporting	BMRS	NA	0.38	0.46	NA	0*	M	AYA	CEBPA	0	0	M2	Relapse
PAXIFA	ROS1	c.350del	p.(Leu117TyrfsTer11)	Frameshift	NM_002944.3	LP	PVS1, PM2 supporting	BMRS	NA	0.45	0.56	0.54	NA	M	Child	KMT2A-MLLT10	0	0.3	Unknown	Induction failure
PAVGBH	RPS27A	c.-17-98T>A	Retention of intron 1 (upstream of start)	Splice	NM_001135592.2	LP	PVS1 (RNA), PM2 supporting	BMRS	NA	0.3	0.38	NA	0.96	M	Infant	None	0	0	M5	Censored

PAXJZX	SBDS	c.258+2T>C	8 bp deletion at the beginning of exon 2	Splice	NM-016038.4	P	PVS1 (RNA), PS3 supporting, PM3 strong	BMRS	NA	0.52	NA	0.55	0.22	F	Child	KMT2A-X	0	3.1	Unknow n	Relapse
PAXJZX	SBDS	c.258+1G>C	8 bp deletion at the beginning of exon 2	Splice	NM-016038.4	P	PVS1 (RNA), PM2 supporting, PM3 supporting	BMRS	NA	0.48	NA	0.47	0.77	F	Child	KMT2A-X	0	3.1	Unknow n	Relapse
PANVPB	SETDB1	c.1214C>A	p.(Ser405Ter)	Stopgain	NM_012432.4	LP	PVS1, PM2 supporting	BMSC	0.6	NA	NA	NA	0*	M	AYA	MLLT10-PSD3	NA	NA	M5	Induction failure
PARXYR	SLX4	c.3895_3896 del	p.(Arg1299Glyf sTer35)	Frameshift	NM_032444.4	LP	PVS1, PM2 supporting	BMSC	0.6	NA	NA	NA	NA	F	AYA	NUP98-NSD1	NA	NA	M1	Relapse
PAUTVU	SLX4	c.928C>T	p.(Arg310Ter)	Stopgain	NM_032444.4	LP	PVS1, PM2 supporting	BMRS	NA	0.58	0.44	NA	0*	F	Infant	RBM15-MRTFA	0	0	M7	Censored
PAVGAM	SMO	c.1921C>G	p.(Pro641Ala)	Missense	NM_005631.5	LP	PS3, PP1, PP3	BMRS	NA	0.44	0.44	0.5	0*	F	Infant	KMT2A-MLLT3	Add(17)(p13) in tumor WGS and not in remission WGS	0	M4	Relapse
PAVVKU	SMO	c.1921C>G	p.(Pro641Ala)	Missense	NM_005631.5	LP	PS3, PP1, PP3	BMRS	NA	0.49	0.42	0.27	0.63	M	Infant	None	0 (t(X;13))	0.2	M7	Relapse
PAVIYH	STXBP2	c.1654G>A	p.(Gly552Ser)	Missense	NM_001272034. 2	LP	PS3, PM3, PP3	BMRS	NA	0.36	0.43	NA	0.46	F	Child	KMT2A-MLLT3	0	0	Unknow n	Censored
PAVDVV	SUFU	c.367C>T	p.(Arg123Cys)	Missense	NM_016169.4	LP	PP1 Strong, PS3 moderate PP3, PM2 supporting	BMRS	NA	0.49	0.52	0.49	0.5	F	AYA	KMT2A-MLLT1	0	0	M5	Relapse
PAXDEG	SYNE1	c.23309T>A	p.(Leu7770Ter)	Stopgain	NM_182961.4	LP	PVS1, PM2 supporting	BMRS	NA	0.71	0.43	0.47	0.56	F	AYA	None	Unable to identify somatic driver	0	M1	Relapse
PAUMMR	TNFRSF13B	c.542C>A	p.(Ala181Glu)	Missense	NM-012452.3	LP	PS3, PS4, PP1, BS1	BMRS	NA	0.43	0.44	NA	0.67*	M	AYA	NUP98-NSD1	0.03	16	Unknow n	Censored
PAUSFM	TNFRSF13B	c.204dup	p.(Leu69ThrfsT er12)	Frameshift	NM-012452.3	P	PVS1, PS4, PP1	BMRS	NA	0.37	0.39	0.44	0*	M	Child	KMT2A-ELL	0	0	M2	Censored

PAUYAE	TNFRSF13B	c.310T>C	p.(Cys104Arg)	Missense	NM-012452.3	LP	PS3, PS4 moderate, PP3, BS2	BMRS	NA	0.68	0.56	NA	0*	F	AYA	NPM1	0	0	M4	Censored
PAUYEV	TNFRSF13B	c.310T>C	p.(Cys104Arg)	Missense	NM-012452.3	LP	PS3, PS4 moderate, PP3, BS2	BMRS	NA	0.48	NA	0.46	1*	M	Child	RUNX1-RUNX1 T1	0	0	Unknow n	Relapse
PAUZSY	TNFRSF13B	c.310T>C	p.(Cys104Arg)	Missense	NM-012452.3	LP	PS3, PS4 moderate, PP3, BS2	BMRS	NA	0.39	NA	0.49	0*	F	Child	None	0 (FLT3-ITD)	0.8	Unknow n	Relapse
PAWUAE	TNFRSF13B	c.310T>C	p.(Cys104Arg)	Missense	NM-012452.3	LP	PS3, PS4 moderate, PP3, BS2	BMRS	NA	0.58	0.63	0.37	0.52*	M	Infant	KMT2A-MLLT10	0	0	Unknow n	Relapse
PAXJZX	TP53	c.626G>A	p.(Arg209Gln)	Missense	NM-001276760. 3	P	PS3, PP3, PP1, PS4 supporting, PM2 supporting	BMRS	NA	0.43	NA	0.75	0.98	F	Child	KMT2A-X	0	3.1	Unknow n	Relapse
PAXLGL	VPS13B	c.5120_5124 del	p.(Lys1707Serf sTer9)	Frameshift	NM_017890.5	LP	PVS1, PM2 supporting	BMRS	NA	0.46	NA	0.52	0.27	M	AYA	KMT2A-ELL	0	0	M2	Relapse
PAVNKI	VPS45	c.695_696del	p.(Tyr232Serfs Ter16)	Frameshift	NM_007259.5	LP	PVS1, PM2 supporting	BMRS	NA	0.36	0.55	0.49	0.48	F	AYA	CBFB-MYH11	0	0	Unknow n	Relapse
PAUTML	XPC	c.463C>T	p.(Arg155Ter)	Stopgain	NM_004628.5	LP	PVS1, PM2 supporting	BMRS	NA	0.37	0.44	NA	NA	NA	NA	NA	0.05 (NRAS G12D)	NA	NA	NA
PASYEJ	XRCC2	c.96del	p.(Phe32Leufs Ter30)	Frameshift	NM_005431.2	LP	PVS1, PM2 supporting	BMSC	0.48	NA	NA	NA	0.2	F	Infant	None	NA	NA	NOS	Induction failure
PAXISI	ZCCHC8	c.317+2T>C	Loss of exon 3	Splice	NM-017612.5	LP	PVS1 (RNA), PM2 supporting	BMRS	NA	0.43	NA	0.5	0.67	M	AYA	CEBPA	0	0	M4	Relapse

USI - unique subject identifier, ACMG - American College of Medical Genetics, AMP - Association of Molecular Pathology, P - pathogenic, LP - likely pathogenic, BMRS - Bone marrow remission sample, BMSC - Bone marrow stromal cells. VAF - variant allele frequency (%), M - male, F - female, FAB - French-American-British subtype. Age groups- Infant: 0-2 years, Child: 3-14 years, AYA: 15-40 years. *Low RNA coverage

Supplementary Table 12: Additional somatic variants in genes with P/LP variants

USI	Gene	Transcript	Variant cDNA	Variant Protein	Diagnosis VAF
PAUUKZ	*FGFR3*	NM_001163213.2	c.1082-510G>A	Splice	0.07
PAUZVP	*PRPF8*	NM_006445.4	c.6929G>A	p.(Arg2310Lys)	0.09
PAUZVP	*PRPF8*	NM_006445.4	c.867-1G>T	Splice	0.06
PAWTWW	*CUX1*	NM_001913.5	c.1823G>A	p.(Gly608Glu)	0.17

Supplementary Table 13: Genes and mode of inheritance included in cohort meta-analysis

Gene	Inheritance
ADA	AR
ADA2	AR
ALAS2	XLD
ANKRD26	AD
APOA1	AD/AR
APOA2	AD/AR
ARID1A	AD
ARID5B	Unknown
ATG2B	Unknown
ATM	AD/AR
BLM	AD/AR
BRCA1	AD
BRCA2	AD
BRIP1	AR
BTK	XLR
CARD11	AD/AR
CASP10	AD
CBL	AD
CD27	AR
CD40LG	XLR
CD70	AR
CDKN2A	AD
CDKN2B	Unknown
CEBPA	AD
CEBPE	AD/AR

CHEK2	AD
CSF3R	AD/AR
CST3	AD
CTC1	AR
CTLA4	AD
CTPS1	AR
CXCR4	AD
DDX41	AD
DIS3	Unknown
DKC1	XLR
DNAJC21	AR
DOCK8	AR
EFTUD1	AR
ELANE	AD
ERCC4	AR
ERCC6L2	AR
ETV6	AD
FADD	AR
FANCA	AR
FANCB	XLR
FANCC	AR
FANCD2	AR
FANCE	AR
FANCF	AR
FANCG	AR
FANCI	AR
FANCL	AR
FANCM	AR
FAS	AD
FASLG	AD
FGA	AD/AR
G6PC3	AR
GATA1	XLR
GATA2	AD
GATA3	AD
GFI1	AD
GSKIP	Unknown

GSN	AD
HAX1	AR
IKZF1	AD
ITK	AR
JAK2	AD
KDM1A	AD
KLHDC8B	AR
LIG4	AR/Smu
LYZ	AD
LZTR1	AD
MAGT1	XLR
MBD4	AD/AR
MECOM	AD
MLH1	AD/AR
MPL	AD/AR/Smu
MRTFA	AR
MSH2	AD/AR
MSH6	AD/AR
NAF1	AD
NBN	AD/AR
NF1	AD
NHP2	AR
NOP10	AR/AD
NPAT	Unknown
NPM1	Smu
PALB2	AR
PARN	AD/AR
PAX5	AD
PGM3	AR
PIK3CD	AD/AR
PIP4K2A	Unknown
PMS2	AD
POT1	AD/AR
PRF1	AR
PTEN	AD
PTPN11	AD
RAD51	AD

RAD51C	AD
RASGRP1	AR
RBBP6	Unknown
RBM8A	AR
RECQL4	AR
RPL11	AD
RPL15	AD
RPL18	AD
RPL23	Unknown
RPL26	AD
RPL27	AD
RPL31	Unknown
RPL35	AD
RPL35A	AD
RPL36	Unknown
RPL5	AD
RPS10	AD
RPS17	AD
RPS19	AD
RPS24	AD
RPS26	AD
RPS27	AD
RPS28	AD
RPS29	AD
RPS7	AD
RTEL1	AD/AR
RUNX1	AD
SAMD9	AD
SAMD9L	AD
SBDS	AR
SH2B3	AR
SH2D1A	XLR
SLX4	AR
SRP54	AD
SRP72	AD
STAT3	AD
STXBP2	AR

TERC	AD
TERF2IP	Unknown
TERT	AD
TET2	AR/Smu
TINF2	AD
TNFRSF13B	AD/AR
TNFRSF9	AR
TP53	AD
TTR	AD
UBE2T	AR
UNC13D	AR
USB1	AR
USP45	AR
VPS45	AR
WAS	XLR
WRAP53	AR
XRCC2	AR
ZNF43	Unknown

Supplementary References:

1. McNeer NA, Philip J, Geiger H, et al. Genetic mechanisms of primary chemotherapy resistance in pediatric acute myeloid leukemia. Leukemia 2019;33(8):1934–43.

2. Li H, Durbin R. Fast and accurate short read alignment with Burrows-Wheeler transform. Bioinformatics 2009;25(14):1754–60.

3. Edmonson MN, Zhang J, Yan C, Finney RP, Meerzaman DM, Buetow KH. Bambino: a variant detector and alignment viewer for next-generation sequencing data in the SAM/BAM format. Bioinformatics 2011;27(6):865–6.

4. Huang YP, Harmon L, Gardner E, et al. bamSliceR: cross-cohort variant and allelic bias analysis for rare variants and rare diseases. bioRxiv [Internet] 2023;Available from: http://dx.doi.org/10.1101/2023.09.15.558026

5. Bolouri H, Ries R, Pardo L, et al. A B-cell developmental gene regulatory network is activated in infant AML. PLoS One 2021;16(11):e0259197.

6. Chen X, Schulz-Trieglaff O, Shaw R, et al. Manta: rapid detection of structural variants and indels for germline and cancer sequencing applications. Bioinformatics 2016;32(8):1220–2.

7. Bolouri H, Farrar JE, Triche T, et al. The molecular landscape of pediatric acute myeloid leukemia reveals

recurrent structural alterations and age-specific mutational interactions. Nat Med 2018;24(1):103–12.

8. Wu T, Hu E, Xu S, et al. clusterProfiler 4.0: A universal enrichment tool for interpreting omics data. Innovation (Camb) 2021;2(3):100141.

9. Friendly M. Visualizing Categorical Data. SAS Institute; 2000.

10. Mayakonda A, Lin D-C, Assenov Y, Plass C, Koeffler HP. Maftools: efficient and comprehensive analysis of somatic variants in cancer. Genome Res 2018;28(11):1747–56.

11. Braunstein EM, Chen H, Juarez F, et al. Germline ERBB2/HER2 coding variants are associated with increased risk of myeloproliferative neoplasms. Cancers (Basel) 2021;13(13):3246.

12. Bao R, Ng A, Sasaki M, et al. Functional common and rare ERBB2 germline variants cooperate in familial and sporadic cancer susceptibility. Cancer Prev Res (Phila) 2021;14(4):441–54.

13. Ju Y, Wang L, Ta S, et al. A germline alteration of ERBB2 increases the risk of breast cancer in Chinese Han women with a familial history of malignant tumors. Oncol Lett 2019;18(3):2885–90.

14. Perkins K, Davis J, Price S, et al. Expanding spectrum of malignancies in ALPS: A cancer predisposing syndrome? Blood 2012;120(21):2149–2149.

15. Shah S, Wu E, Rao VK, Tarrant TK. Autoimmune lymphoproliferative syndrome: an update and review of the literature. Curr Allergy Asthma Rep 2014;14(9):462.

16. Rio-Machin A, Vulliamy T, Hug N, et al. The complex genetic landscape of familial MDS and AML reveals pathogenic germline variants. Nat Commun 2020;11(1):1044.

17. Molteni E, Bono E, Gallì A, et al. Prevalence and clinical expression of germ line predisposition to myeloid neoplasms in adults with marrow hypocellularity. Blood 2023;142(7):643–57.

18. Hug N, Aitken S, Longman D, et al. A dual role for the RNA helicase DHX34 in NMD and pre-mRNA splicing and its function in hematopoietic differentiation. RNA 2022;28(9):1224–38.

19. Chaudhry MS, Gilmour KC, House IG, et al. Missense mutations in the perforin (PRF1) gene as a cause of hereditary cancer predisposition. Oncoimmunology 2016;5(7):e1179415.

20. Cannella S, Santoro A, Bruno G, et al. Germline mutations of the perforin gene are a frequent occurrence in childhood anaplastic large cell lymphoma. Cancer 2007;109(12):2566–71.

21. Grisanti K, Patadia D, Scherzer R. P195 Manifestation of hemophagocytic lymphohistiocytosis in an adolescent with a heterozygous PRF1 mutation. Ann Allergy Asthma Immunol 2016;117(5):S80.

22. Xin X, Wang N, Zhang Y. Hemophagocytic lymphohistiocytosis with a hemizygous PRF1 c.674G>A mutation. Am J Med Sci 2023;366(5):387–94.

23. McReynolds LJ, Giri N, Leathwood L, Risch MO, Carr AG, Alter BP. Risk of cancer in heterozygous relatives of patients with Fanconi anemia. Genet Med 2022;24(1):245–50.

24. Nie D, Zhang J, Wang F, et al. Fanconi anemia gene-associated germline predisposition in aplastic anemia and hematologic malignancies. Front Med 2022;16(3):459–66.

25. Tischkowitz MD, Morgan NV, Grimwade D, et al. Deletion and reduced expression of the Fanconi anemia FANCA gene in sporadic acute myeloid leukemia. Leukemia 2004;18(3):420–5.

26. Abbasi S, Rasouli M. A rare FANCA gene variation as a breast cancer susceptibility allele in an Iranian population. Mol Med Rep 2017;15(6):3983–8.

27. Xia Q, Zhao L-Y, Yan Y-D, Liao Y, Di Y-S, Xiao X-Y. A multiple primary malignancy patient with FANCA gene mutation: A case report and literature review. Front Oncol 2020;10:1199.

28. Recommendations for Preventive Care for Women with Rare Genetic Cause of Breast and Ovarian Cancer » Klinicka onkologie Journal » Linkos.cz [Internet]. [cited 2025 Feb 7];Available from: https://www.linkos.cz/english-summary/klinicka-onkologie-journal/2019-08-18-supplementum-2-en/doporuceni-pro-sledovani-zen-se-vzacnejsimi-genetickymi-pricinami-nadoru-prsu-a-1/

29. Chen P-C, Yin J, Yu H-W, et al. Next-generation sequencing identifies rare variants associated with Noonan syndrome. Proc Natl Acad Sci U S A 2014;111(31):11473–8.

30. Arafeh R, Qutob N, Emmanuel R, et al. Recurrent inactivating RASA2 mutations in melanoma. Nat Genet 2015;47(12):1408–10.

31. Olkinuora A, Nieminen TT, Douglas S, et al. Identification of DHX40 as a candidate susceptibility gene for colorectal and hematological neoplasia. Leukemia 2023;37(11):2301–5.

32. Yang F, Long N, Anekpuritanang T, et al. Identification and prioritization of myeloid malignancy germline variants in a large cohort of adult patients with AML. Blood 2022;139(8):1208–21.

33. Singh M, Chaudhry P, Merchant AA. Primary cilia are present on human blood and bone marrow cells and mediate Hedgehog signaling. Exp Hematol 2016;44(12):1181–7.e2.

34. Kang MR, Kim MS, Oh JE, et al. Frameshift mutations of autophagy-related genes ATG2B, ATG5, ATG9B and ATG12 in gastric and colorectal cancers with microsatellite instability: ATG gene mutations. J Pathol 2009;217(5):702–6.

35. Yu T, Ben S, Ma L, et al. Genetic variants in autophagy-related gene ATG2B predict the prognosis of colorectal cancer patients receiving chemotherapy. Front Oncol 2022;12:876424.

36. Park JW, Kim Y, Lee S-B, et al. Autophagy inhibits cancer stemness in triple-negative breast cancer via miR-181a-mediated regulation of ATG5 and/or ATG2B. Mol Oncol 2022;16(9):1857–75.

37. Tsao AS, Wistuba I, Xia D, et al. Germline and somatic smoothened mutations in non-small-cell lung cancer are potentially responsive to hedgehog inhibitor vismodegib. JCO Precis Oncol 2017;1(1):1–10.

38. Isidori F, Bozzarelli I, Ferrari S, et al. RASAL1 and ROS1 gene variants in hereditary BReast CAncer. Cancers (Basel) 2020;12(9):2539.

39. de Garibay GR, Díaz A, Gaviña B, et al. Low prevalence of SLX4 loss-of-function mutations in non-BRCA1/2 breast and/or ovarian cancer families. Eur J Hum Genet 2013;21(8):883–6.

40. Shah S, Kim Y, Ostrovnaya I, et al. Assessment of SLX4 Mutations in Hereditary Breast Cancers. PLoS One 2013;8(6):e66961.

41. Neidhardt G, Hauke J, Ramser J, et al. Association between loss-of-function mutations within the FANCM gene and early-onset familial breast cancer. JAMA Oncol 2017;3(9):1245–8.

42. Figlioli G, Bogliolo M, Catucci I, et al. The FANCM:p.Arg658* truncating variant is associated with risk of triple-negative breast cancer. NPJ Breast Cancer 2019;5(1):38.

43. Tavera-Tapia A, de la Hoya M, Calvete O, et al. RECQL5: Another DNA helicase potentially involved in hereditary breast cancer susceptibility: TAVERA-TAPIAet al. Hum Mutat 2019;40(5):566–77.

44. He Y-J, Qiao Z-Y, Gao B, Zhang X-H, Wen Y-Y. Association between RECQL5 genetic polymorphisms and susceptibility to breast cancer. Tumour Biol 2014;35(12):12201–4.

45. Shen W, Kerr CM, Przychozen B, et al. Impact of germline CTC1 alterations on telomere length in

acquired bone marrow failure. Br J Haematol 2019;185(5):935–9.

46. Fan C, Zhang J, Ouyang T, et al. RAD50 germline mutations are associated with poor survival in BRCA1/2-negative breast cancer patients. Int J Cancer 2018;143(8):1935–42.

47. Stastna B, Dolezalova T, Matejkova K, et al. Germline pathogenic variants in the MRE11, RAD50, and NBN (MRN) genes in cancer predisposition: A systematic review and meta-analysis. Int J Cancer 2024;155(9):1604–15.

48. Kluźniak W, Wokołorczyk D, Rusak B, et al. Inherited variants in XRCC2 and the risk of breast cancer. Breast Cancer Res Treat 2019;178(3):657–63.

49. Win AK, Cleary SP, Dowty JG, et al. Cancer risks for monoallelic MUTYH mutation carriers with a family history of colorectal cancer. Int J Cancer 2011;129(9):2256–62.

50. Win AK, Reece JC, Dowty JG, et al. Risk of extracolonic cancers for people with biallelic and monoallelic mutations in MUTYH. Int J Cancer 2016;139(7):1557–63.

51. Zhu M, Chen X, Zhang H, et al. AluYb8 insertion in the MUTYH gene and risk of early-onset breast and gastric cancers in the Chinese population. Asian Pac J Cancer Prev 2011;12(6):1451–5.

52. Rennert G, Lejbkowicz F, Cohen I, Pinchev M, Rennert HS, Barnett-Griness O. MutYH mutation carriers have increased breast cancer risk. Cancer 2012;118(8):1989–93.

53. Barreiro RAS, Sabbaga J, Rossi BM, et al. Monoallelic deleterious MUTYH germline variants as a driver for tumorigenesis. J Pathol 2022;256(2):214–22.

54. Li N, Zethoven M, McInerny S, et al. Evaluation of the association of heterozygous germline variants in NTHL1 with breast cancer predisposition: an international multi-center study of 47,180 subjects. NPJ Breast Cancer 2021;7(1):52.

55. Maciaszek JL, Oak N, Chen W, et al. Enrichment of heterozygous germline RECQL4 loss-of-function variants in pediatric osteosarcoma. Cold Spring Harb Mol Case Stud 2019;5(5):a004218.

56. Martin-Giacalone BA, Rideau T-T, Scheurer ME, Lupo PJ, Wang LL. Cancer risk among RECQL4 heterozygotes. Cancer Genet 2022;262-263:107–10.

57. Francisco G, Menezes PR, Eluf-Neto J, Chammas R. XPC polymorphisms play a role in tissue-specific carcinogenesis: a meta-analysis. Eur J Hum Genet 2008;16(6):724–34.

58. Zhou H, Saliba J, Sandusky GE, Sears CR. XPC protects against smoking- and carcinogen-induced lung adenocarcinoma. Carcinogenesis 2019;40(3):403–11.

59. Strom SS, Estey E, Outschoorn UM, Garcia-Manero G. Acute myeloid leukemia outcome: role of nucleotide excision repair polymorphisms in intermediate risk patients. Leuk Lymphoma 2010;51(4):598–605.

60. Liu C, Zhao L, Zhao J, Xu Q, Song Y, Wang H. Decreased ADAMTS-13 level is related to inflammation factors and risk stratification of acute lymphoblastic leukemia patients. Medicine (Baltimore) 2017;96(7):e6136.

61. Liu C, Han M, Zhao L, et al. ADAMTS-13 activity reduction in plasma of acute myeloid leukemia predicts poor prognosis after bone marrow transplantation. Hematology 2019;24(1):129–33.

62. Taddei TH, Kacena KA, Yang M, et al. The underrecognized progressive nature of N370S Gaucher disease and assessment of cancer risk in 403 patients. Am J Hematol 2009;84(4):208–14.

63. Qiu Z, Wang X, Yang Z, et al. GBA1-dependent membrane glucosylceramide reprogramming promotes

liver cancer metastasis via activation of the Wnt/β-catenin signalling pathway. Cell Death Dis 2022;13(5):508.

64. Aly M, Ramdzan ZM, Nagata Y, et al. Distinct clinical and biological implications of CUX1 in myeloid neoplasms. Blood Adv 2019;3(14):2164–78.

65. Oppermann H, Marcos-Grañeda E, Weiss LA, et al. CUX1-related neurodevelopmental disorder: deep insights into phenotype-genotype spectrum and underlying pathology. Eur J Hum Genet 2023;31(11):1251–60.

66. Huang D, Nagata Y, Grossmann V, et al. BRCC3 mutations in myeloid neoplasms. Haematologica 2015;100(8):1051–7.

67. Houzelstein D, Bullock SL, Lynch DE, Grigorieva EF, Wilson VA, Beddington RSP. Growth and early postimplantation defects in mice deficient for the bromodomain-containing protein Brd4. Mol Cell Biol 2002;22(11):3794–802.

68. Moura S, Hartl I, Brumovska V, et al. Exploring FGFR3 mutations in the male germline: Implications for clonal germline expansions and paternal age-related dysplasias. Genome Biol Evol 2024;16(2):evae015.

69. Helgason CD, Damen JE, Rosten P, et al. Targeted disruption of SHIP leads to hemopoietic perturbations, lung pathology, and a shortened life span. Genes Dev 1998;12(11):1610–20.

70. Galvin RT, Zheng C, Fitzpatrick G, et al. MYO5A::FGFR1 represents a novel fusion event in pediatric low-grade glioma. Neurooncol Adv 2023;5(1):vdad017.

71. Alves CP, Moraes MH, Sousa JF, et al. Myosin-Va contributes to manifestation of malignant-related properties in melanoma cells. J Invest Dermatol 2013;133(12):2809–12.

72. Bonato A, Chakraborty S, Bomben R, et al. NFKBIE mutations are selected by the tumor microenvironment and contribute to immune escape in chronic lymphocytic leukemia. Leukemia 2024;38(7):1511–21.

73. Lazaro-Camp VJ, Salari K, Meng X, Yang S. SETDB1 in cancer: overexpression and its therapeutic implications. Am J Cancer Res 2021;11(5):1803–27.
